# RECOMBINE identifies recurrent composite markers of cell types and states

**DOI:** 10.1101/gr.280817.125

**Published:** 2026-06

**Authors:** Xubin Li, Justin Nguyen, Anil Korkut

**Affiliations:** Department of Bioinformatics and Computational Biology, The University of Texas MD Anderson Cancer Center, Houston, Texas 77030, USA

## Abstract

Biological function is mediated by the hierarchical organization of cell types and states within tissue ecosystems. Identifying interpretable composite marker sets that both define and distinguish hierarchical cell identities is essential for decoding biological complexity yet remains a major challenge. Here, we present RECOMBINE, an algorithm that identifies recurrent composite marker sets to define hierarchical cell identities. Validation using both simulated and biological data sets demonstrates that RECOMBINE is robust to hyperparameter variation and data sparsity, and achieves higher accuracy in identifying discriminant markers compared with existing approaches. As a partition-free framework, RECOMBINE is particularly powerful for data sets characterized by continuous cell-state transitions, in which defining discrete boundaries is inappropriate. This capability is demonstrated by its application to zebrafish development, revealing gradual transcriptional transitions across embryonic stages, and to the mouse cerebellum, in which it uncovers transcriptional variation shaped by spatial gradients. When applied to single-cell data and validated with spatial transcriptomic data from the mouse visual cortex, RECOMBINE identifies key cell-type markers and generates a robust gene panel for targeted spatial profiling. It also uncovers markers of CD8^+^ T cell states, including *GZMK*^+^*HAVCR2*^−^ effector memory cells associated with anti-PD-1 therapy response. Finally, using data from the Tabula Sapiens project, RECOMBINE identifies composite marker sets across a broad range of human tissues. Together, these results highlight RECOMBINE as a robust, data-driven framework for optimized marker selection, enabling the discovery and validation of hierarchical cell identities across diverse tissue contexts.

Tissue ecosystems comprise hierarchical cell types and dynamic states that underpin biological structure and function (Zhang et al. 2018; Osumi-Sutherland et al. 2021). Characterizing these cell types and states has been a fundamental goal in biology. A key challenge lies in identifying a minimal set of composite markers capable of distinguishing hierarchical cell identities, particularly when these identities are in a functional continuum with overlapping molecular features (Becker et al. 2022; Kong et al. 2022; Lange et al. 2022). Such concise, composite markers, which capture the drivers of tissue heterogeneity, can facilitate the prediction of healthy and aberrant phenotypes, including immune states, developmental stages, and therapeutic responses.

Advances in single-cell transcriptomics have revealed the intricate cellular composition and organization of tissue ecosystems in both healthy and diseased contexts (Aldridge and Teichmann 2020; Longo et al. 2021; Rao et al. 2021; Elmentaite et al. 2022; Liu and Zhang 2022). However, robust analytical approaches are needed to decode the composite markers that drive cellular heterogeneity and function within tissues. In parallel, the rise of targeted spatial transcriptomic technologies, including MERFISH (Moffitt et al. 2018), STARmap (Wang et al. 2018), and CosMx (He et al. 2022), highlights the necessity of preselected marker panels to distinguish diverse cell types and states within tissues. Such marker panels are also critical for developing cost-effective clinical assays that accurately reflect biological processes underlying disease mechanisms (Gohil et al. 2021). Despite this pressing need, the identification of concise, composite markers capable of optimally discriminating hierarchical cell subpopulations remains a significant challenge.

Current computational approaches for identifying markers of cell subpopulations can be broadly categorized into differentially expressed gene (DEG)–based and non-DEG-based methods. DEG-based approaches typically rely on partitional clustering to group cells into discrete clusters, followed by statistical testing to identify DEGs that distinguish each cluster from others (Luecken and Theis 2019; Hao et al. 2021). However, these DEG lists are often lengthy and challenging to interpret. Moreover, the selected markers are inherently biased by predefined cluster labels and fail to capture dynamic cell states within clusters. In contrast, non-DEG-based methods do not depend on discrete partitions. Common examples include the selection of highly variable genes (HVGs) ranked by standardized variance derived from the mean–variance relationship, as well as principal component analysis (PCA) loadings ranked by their contribution to total variance. Although computationally efficient, these approaches are largely heuristic, focusing on feature variability rather than discrimination and thus cannot effectively distinguish both discrete and continuous cell identities. This limitation highlights a critical challenge in single-cell analysis: the ability to flexibly define varying levels of granularity for mapping cell identities (Lähnemann et al. 2020). Recently, partition-free hierarchical methods have shown promise in addressing this challenge. For instance, MetaCell (Baran et al. 2019) and SEACells (Persad et al. 2023) create metacells that represent highly granular and distinct cell states, whereas Milo leverages *k*-nearest neighbors graph representations to identify perturbed cell states obscured by partitional clustering (Dann et al. 2022). Despite these advances, a partition-free method capable of optimally selecting composite markers to characterize hierarchical cell identities remains an unmet need.

To address this challenge, we developed a novel computational framework, recurrent composite markers for biological identities with neighborhood enrichment (RECOMBINE), designed to identify optimized, composite marker sets that distinguish hierarchical cell subpopulations. We assessed RECOMBINE's performance using both simulated and biological data sets, demonstrating that RECOMBINE is robust to hyperparameter variation and data sparsity and outperformed other feature selection methods. Applications of RECOMBINE to single-cell and spatial transcriptomic data underscore its robustness and versatility in optimizing marker selection for hierarchical cell identities within complex tissue ecosystems.

## Results

### RECOMBINE identifies composite marker sets for hierarchical cell identities

**Figure 1.**
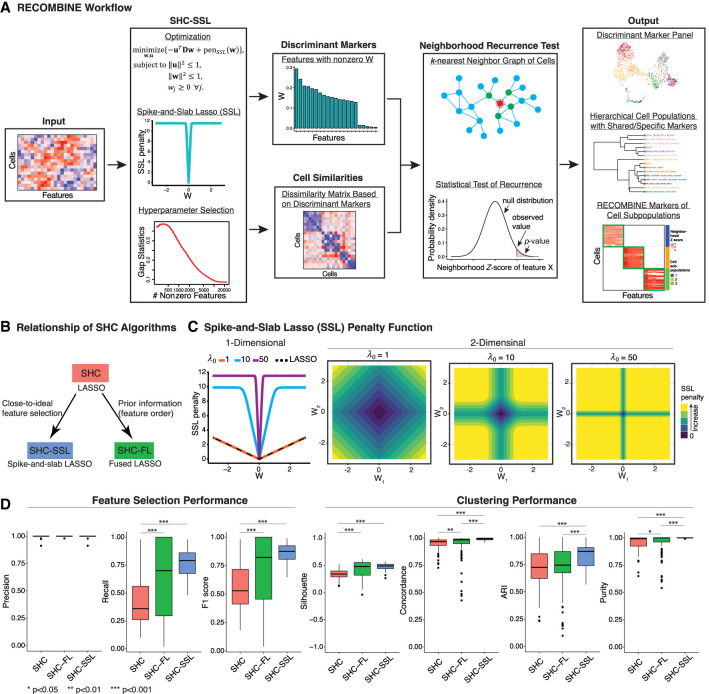
RECOMBINE is a computational framework that determines recurrent composite markers of cell types and states. (*A*) RECOMBINE workflow. (*B*) Relationship of sparse hierarchical clustering algorithms. (*C*) The SSL penalty function. The SSL penalty as a function of **w** with λ_1_ = 0.001 and λ_0_ ∈ {1, 10, 50}, where **w** is a one-dimensional (*left* panel) or two-dimensional variable (other panels). The SSL penalty converges to the LASSO penalty with decreasing λ_0_ and to the L0-norm penalty with increasing λ_0_. Therefore, the SSL penalty forms a data-adjustable bridge between an L0-norm and a LASSO penalty, which empowers SHC-SSL without any a priori information. (*D*) Benchmarking of the sparse hierarchical clustering algorithms for feature selection precision and recall, as well as clustering performance, using 100 randomly generated simulation data sets. Silhouette measures consistency of the dissimilarity matrix based on discriminant features and ground-truth labels of clusters; concordance measures clustering performance after hierarchical clustering; and the adjusted Rand index (ARI) and purity measure clustering performance after Leiden clustering. For each metric, a higher value is better.

RECOMBINE is a computational framework designed for unbiased selection of composite markers to distinguish hierarchical cell subpopulations (Fig. 1A). It processes high-dimensional data, such as single-cell transcriptomics, to identify markers that delineate cell populations into hierarchical subgroups using the sparse hierarchical clustering with the spike-and-slab LASSO (SHC-SSL) algorithm, which we have developed in this study. The SHC-SSL step selects discriminant markers with nonzero weights, representing features that most strongly separate hierarchical subpopulations. Building on these features, RECOMBINE identifies recurrent composite markers at the single-cell level through the construction of *k*-nearest neighbors graphs, followed by a neighborhood recurrence test. A neighborhood *Z*-score is calculated for each discriminant marker, reflecting the relative marker expression across all neighborhoods. Marker significance is determined using *P*-values derived from these *Z*-scores with multiple-testing correction. A marker is defined as recurrent for a given cell subpopulation if it meets two criteria: a high fraction of significant cells and an elevated mean neighborhood *Z*-score. The outputs of RECOMBINE include (1) a panel of discriminant markers that optimally separate all cells in the data set and (2) recurrent composite markers that are specific to individual or shared cell subpopulations. Whereas discriminant markers represent a globally optimized gene set that defines overall cell-type and cell-state distribution, recurrent composite markers capture subpopulation-specific or cross-subpopulation signatures.

Using simulation data, we systematically benchmarked the performance of SHC-SSL against two other sparse hierarchical clustering algorithms: the conventional sparse hierarchical clustering with regular LASSO (SHC) (Witten and Tibshirani 2010) and a newly developed algorithm, SHC with fused LASSO (SHC-FL) (Fig. 1B). The SSL penalty in SHC-SSL provides a continuum between LASSO and L0-norm, enabling more precise selection of discriminant features compared with the LASSO used in SHC (Fig. 1C). SHC-FL incorporates prior information about feature order through the fused LASSO (Methods). All three algorithms achieved median precisions of 100%, indicating that the identified features were true discriminant markers (Fig. 1D). SHC-SSL demonstrated the highest median recall and F1 score with minimal variation, reflecting superior feature selection accuracy and a low false-negative rate (Fig. 1D). Incorporating prior information through SHC-FL significantly improved its feature selection performance. However, SHC-FL exhibited high recall variability (ranging from 20% to 100%), suggesting strong dependence on the alignment of prior information with the data. In contrast, SHC consistently showed lower recall and F1 scores compared with the other algorithms. To evaluate clustering performance, we assessed four metrics: silhouette score, concordance, adjusted Rand index, and purity (Methods). Across all metrics, performance scores increased monotonically from SHC to SHC-FL to SHC-SSL, with SHC-SSL exhibiting the best median performance and the least variation (Fig. 1D). These findings demonstrate that SHC-SSL is the most accurate and robust algorithm for both feature selection and clustering among the tested sparse hierarchical clustering methods.

### RECOMBINE is robust to hyperparameter variation and data sparsity, and outperforms other feature selection methods

**Figure 2.**
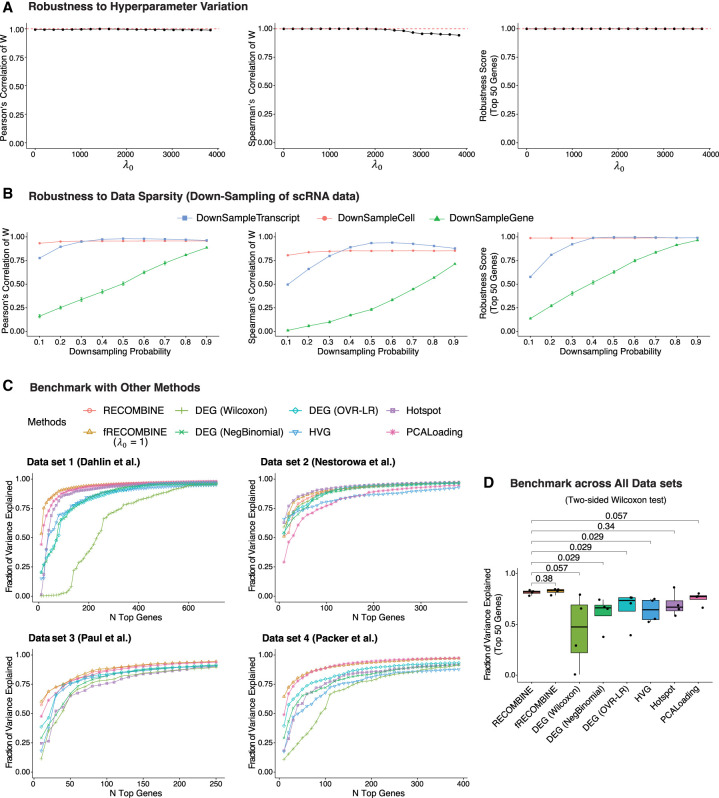
Benchmarking RECOMBINE's robustness and performance relative to other methods. (*A*) Robustness to hyperparameter variation was evaluated by comparing the gene weights (W) obtained through varying λ_0_ to the reference weights (W_ref_) derived from the optimal λ_0_ selected via the gap statistic. Robustness was quantified using the Pearson's correlation (*left*), Spearman's correlation (*middle*), and the robustness score of the top 50 genes ranked by W relative to those ranked by W_ref_ (for details, see Methods). (*B*) Robustness to scRNA-seq data sparsity was assessed by comparing W from downsampled data sets with W_ref_ from the complete data set. Three types of downsampling were performed: transcript level, cell level, and gene level. (*C*) Comparison of marker selection among RECOMBINE, fRECOMBINE (λ_0_ = 1), and other approaches including DEG (Wilcoxon test), DEG (negative binomial test), DEG (OVR-LR), HVG, Hotspot, and PCA loadings. For each method, the number of selected markers was matched to that identified by RECOMBINE. The fraction of variance explained by each method represents the proportion of total variance captured in the top 10 principal components of the complete data set with all genes. The RECOMBINE and fRECOMBINE curves are indistinguishable in data sets 1 and 4, as both methods exhibit identical performance. (*D*) Fraction of variance explained by the top 50 genes from each method across all data sets. (DEG) Differentially expressed genes; (OVR-LR) one-versus-rest logistic regression; (HVG) highly variable genes.

We systematically benchmarked RECOMBINE's performance across multiple biological data sets and conditions. We first assessed RECOMBINE's robustness to hyperparameter variation using a single-cell RNA-seq data set of murine hematopoiesis (Dahlin et al. 2018). RECOMBINE has two hyperparameters, λ_0_ and λ_1_, where λ_0_ > λ_1_. Increasing the difference between λ_0_ and λ_1_ results in a smaller number of selected features. In practice, λ_1_ is fixed, and an optimal λ_0_ is determined by maximizing the gap statistic, which quantifies the hierarchical clustering strength of features selected by RECOMBINE relative to random features of equal size. The optimal λ_0_ thus defines the optimal set of features with nonzero weights. To assess the effect of λ_0_ variation, we fixed λ_1_ = 0.0001 and varied λ_0_ from 0.001 to 4000. We then compared the feature weights (W) obtained from each λ_0_ to those from the optimal λ_0_ (W_ref_) (Fig. 2A). The Pearson's correlation between W and W_ref_ remained extremely high, and their Spearman's correlation exceeded 0.9 even at λ_0_ = 4000, staying near one for λ_0_ < 2000. The top 50 features ranked by weight were nearly identical across all the λ_0_ values tested, demonstrating that RECOMBINE's top-ranked features are highly stable. Thus, small or moderate λ_0_ values yield feature sets that are nearly indistinguishable from the optimal one. Although a high λ_0_ produces a slightly different set of selected features, the top features remain highly stable.

Next, we evaluated RECOMBINE's robustness to data sparsity by performing three types of downsampling on the same data set: transcript level, cell level, and gene level (Fig. 2B). At the transcript level, downsampling probabilities greater than 0.2 had minimal effect on feature weights or rankings; the top 50 features remained almost identical to those from the complete data. At the cell level, RECOMBINE remained exceptionally robust: Even at a downsampling probability of 0.1, the feature-weight correlation closely matched that of the full data set, and the top 50 features were identical. This robustness arises from RECOMBINE's pseudocell strategy, which aggregates transcriptionally similar cells, stabilizing variance across cells in the data set and reducing sensitivity to sparsity. At the gene level, feature-weight correlation and recovery of top features decreased approximately linearly with the proportion of missing genes, indicating that RECOMBINE robustly identifies informative genes from whatever subset is present. Collectively, RECOMBINE demonstrates strong robustness to transcript-, cell-, and gene-level sparsity.

Finally, we benchmarked RECOMBINE's performance against other feature selection methods. Because RECOMBINE is robust to hyperparameter variation, we also implemented a fixed hyperparameter version, fRECOMBINE, with λ_1_ = 0.0001 and λ_0_ = 1. Without hyperparameter optimization, fRECOMBINE achieves comparable performance while being two to three orders of magnitude faster than RECOMBINE, making it ideal for rapid feature weighting and ranking. RECOMBINE, in contrast, is best suited for identifying the optimal number of discriminant features with nonzero weights. We compared RECOMBINE and fRECOMBINE with six established methods: three DEG-based approaches (Wilcoxon test, negative binomial test, and one-vs.-rest logistic regression [OVR-LR]) and three non-DEG-based methods (HVGs, Hotspot [DeTomaso and Yosef 2021], and PCA loadings). Benchmarking was performed using three murine hematopoiesis data sets (Paul et al. 2015; Nestorowa et al. 2016; Dahlin et al. 2018) and one *Caenorhabditis elegans* embryogenesis data set (Supplemental Fig. S1; Packer et al. 2019). For each data set, RECOMBINE selected an optimized feature set, and all other methods were constrained to select the same number of features. For DEG-based methods, genes with log_2_-fold-change >0.25 and adjusted *P* < 0.05 were retained. Within each cluster, genes were ranked by adjusted *P*-value (ties broken by descending fold-change), and a combined DEG list was created by evenly selecting top markers from each cluster to match the RECOMBINE marker count. For non-DEG-based methods, features were ranked by standardized variance of HVGs, Hotspot *Z*-scores, or the sum of absolute PCA loadings, and the top features were selected accordingly. Across all data sets, RECOMBINE and fRECOMBINE consistently ranked first or second (Fig. 2C; Supplemental Fig. S2) and significantly outperformed other methods overall (Fig. 2D). Although Hotspot (DeTomaso and Yosef 2021), a graph-based autocorrelation method, achieved the highest performance in data set 2, it performed inconsistently across the remaining data sets, displaying high variability (Fig. 2D). In contrast, RECOMBINE and fRECOMBINE maintained high performance with minimal variation. In summary, RECOMBINE and fRECOMBINE demonstrate robustness to both hyperparameter variation and data sparsity, exhibiting superior and stable performance across diverse data sets.

### RECOMBINE identifies discriminant markers of cell-state transitions in continuum

Unlike DEG-based methods that rely on discrete cell partitions, RECOMBINE is a partition-free framework that selects discriminant markers jointly with hierarchical clustering of cells. Consequently, RECOMBINE markers form an optimized feature set that captures the cell-state hierarchy, making RECOMBINE particularly powerful for data sets characterized by continuous transitions, in which defining discrete boundaries between cell states is challenging.

**Figure 3.**
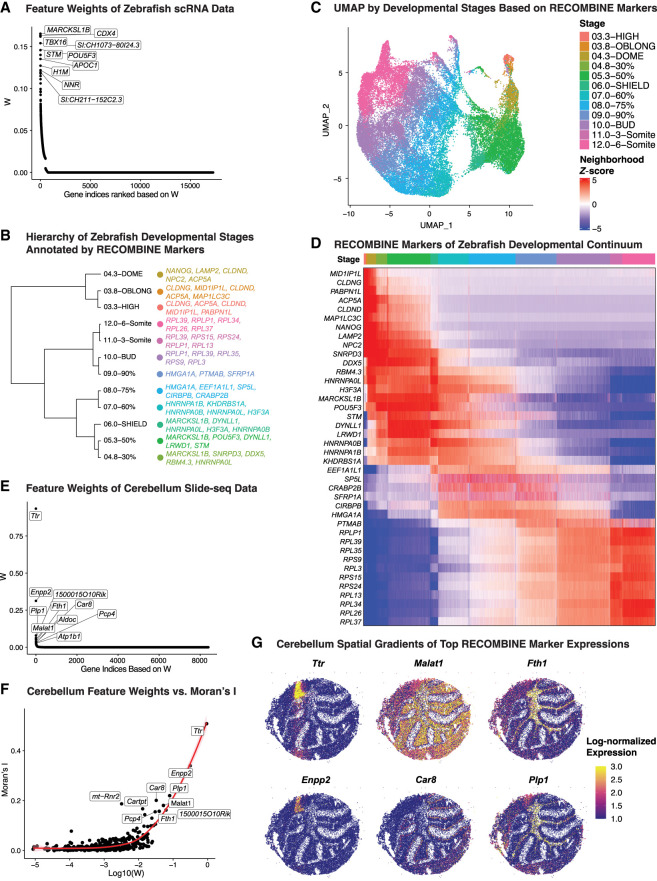
RECOMBINE identifies discriminant markers of transcriptional continuums in zebrafish development and spatial gradients in the mouse cerebellum. (*A*–*D*) Zebrafish developmental continuums. (*A*) Feature weights of all genes, with the top 10 discriminant markers highlighted. (*B*) Hierarchical tree of developmental stages based on RECOMBINE markers and annotated by the top five markers. (*C*) UMAP embedding based on RECOMBINE markers, colored by developmental stage. (*D*) Heatmap of neighborhood *Z*-scores illustrating transcriptional continuums of RECOMBINE gene modules across developmental stages. (*E*–*G*) Mouse cerebellum spatial gradients. (*E*) Feature weights of all genes, with the top 10 discriminant markers labeled. (*F*) Correlation between RECOMBINE feature weights and Moran's *I*, a measure of spatial autocorrelation. (*G*) Spatial maps depicting expression gradients of top RECOMBINE markers.

To demonstrate RECOMBINE's ability to capture transcriptional continuums, we applied it to a single-cell RNA-seq data set of zebrafish embryogenesis spanning 12 developmental stages (Farrell et al. 2018). From the expression of 17,239 genes across 38,731 cells, RECOMBINE selected 756 discriminant markers with nonzero weights (Fig. 3A; Supplemental Table S1). Marker weights were independent of mean expression levels, capturing both highly and lowly expressed genes (Supplemental Fig. S3A). Hierarchical clustering of cells based on these markers aligned precisely with developmental progression from 3.3 to 12 h postfertilization (Fig. 3B). UMAP embedding further revealed a smooth developmental trajectory and within-stage transcriptional gradients (Fig. 3C). The expression of RECOMBINE-selected genes varied continuously across developmental time, suggesting dynamic regulatory programs driving embryogenesis (Fig. 3D). Thus, RECOMBINE identified a concise set of discriminant markers that reconstructed the developmental hierarchy, captured gradual transcriptional transitions, and revealed gene modules potentially regulating embryonic progression.

Beyond temporal dynamics, RECOMBINE can also delineate spatially continuous cell-state variation. To illustrate this, we applied it to a Slide-seq data set of the mouse cerebellum (Rodriques et al. 2019). From the expression of 8401 genes across 35,979 spatially indexed libraries, each representing a small cluster of neighboring cells, RECOMBINE identified 426 discriminant markers spanning both highly and lowly expressed genes (Fig. 3E; Supplemental Fig. S3B; Supplemental Table S2). Their feature weights showed a strong correlation with Moran's *I*, a measure of spatial autocorrelation (Fig. 3F). Genes with large feature weights exhibited high Moran's *I*-values, indicating that transcriptional variation is closely linked to spatial organization. Indeed, top RECOMBINE markers such as *Ttr*, *Enpp2*, *Malat1*, *Car8*, *Fth1*, and *Plp1* displayed smooth spatial gradients across cerebellar regions (Fig. 3G). Together, these results demonstrate that RECOMBINE effectively identifies transcriptional markers underlying both developmental and spatial continuums of cell states.

### RECOBMINE optimizes marker panels for targeted spatial transcriptomics

Targeted spatial transcriptomic technologies have enabled spatially resolved molecular profiling at single-cell resolution (Moffitt et al. 2018; Wang et al. 2018; He et al. 2022). However, these technologies require the prior selection of a gene panel capable of discriminating diverse cell types and states. To address this challenge, we utilized RECOMBINE, which selects markers from scRNA-seq data without any prior knowledge of cell-type content. We applied RECOMBINE to an scRNA-seq data set of the mouse visual cortex (Tasic et al. 2018) and validated the selected markers using a STARmap spatial transcriptomic data set of the same tissue that was obtained independently (Wang et al. 2018).

**Figure 4.**
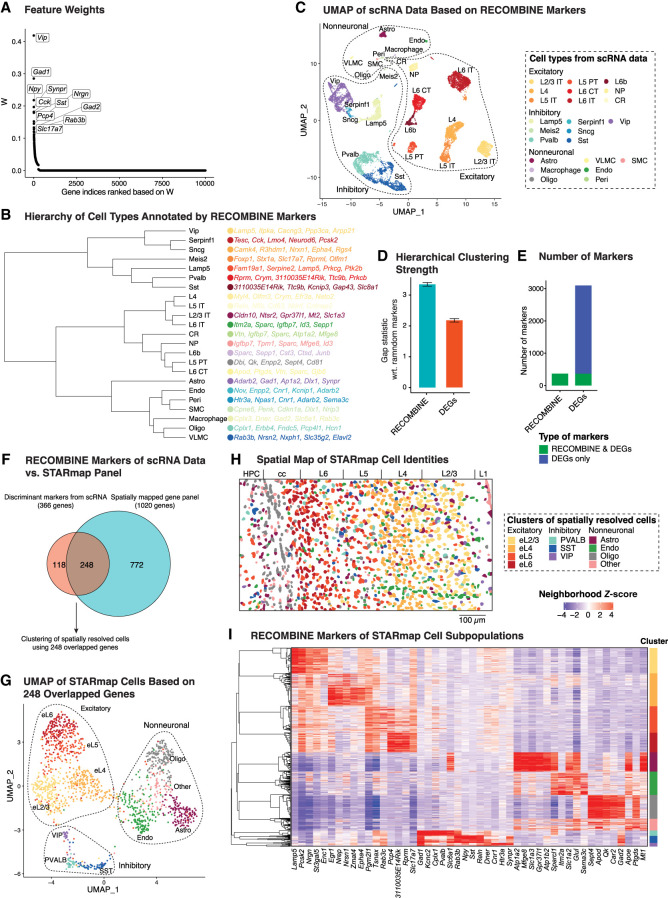
RECOMBINE selects composite markers that define hierarchical cell types and provides an optimized panel for spatial profiling of mouse visual cortex. (*A*) Feature weights of all genes in which the top 10 discriminant markers are labeled. (*B*) Hierarchy of cell types annotated by top five RECOMBINE markers. (*C*) UMAP of cells based on RECOMBINE markers, in which colors indicate cell-type labels from Tasic et al. (2018). Cell-type abbreviations: (IT) intratelencephalic, (PT) pyramidal tract, (CT) corticothalamic, (NP) near-projecting, (CR) Cajal–Retzius cell, (Astro) astrocyte, (Oligo) oligodendrocyte, (VLMC) vascular leptomeningeal cell, (Endo) endothelial cell, (Peri) pericyte, and (SMC) smooth muscle cell. (*D*) Comparison of hierarchical clustering performance between RECOMBINE markers and top DEGs of the same size. (*E*) Comparison of RECOMBINE markers and DEGs. DEGs were obtained by Wilcoxon tests of each cell type with respect to the rest of cell types and filtered based on an absolute log_2_ fold-change >0.25 and false-discovery rate <0.05. (*F*) Venn diagram of discriminant markers in the scRNA data and the genes profiled in the spatial data. (*G*) UMAP of spatially resolved cells based on RECOMBINE markers (N = 248) and colored by clusters. (*H*) Spatial map of cells with the same color code of cell clusters in *E*. (*I*) Heatmap of neighborhood *Z*-scores showing gene modules across cell clusters of the spatial data. For each cluster, the gene module includes the top five genes with mean neighborhood *Z*-score > 2, ranked by decreasing fraction of significant cells. (L1–L6) The six neocortical layers, (cc) corpus callosum, and (HPC) hippocampus.

The scRNA-seq data set included 14,249 cells representing 23 cell types across three major compartments: excitatory neurons, inhibitory neurons, and nonneuronal cells. RECOMBINE identified 366 discriminant genes (Fig. 4A; Supplemental Fig. S4A; Supplemental Table S3), including key markers for inhibitory neurons (e.g., *Gad1*, *Gad2*, *Vip*, *Npy*, *Synpr*, *Cck*, *Sst*, and *Rab3b*) and excitatory neurons (e.g., *Slc17a7*, *Pcp4*, and *Nrgn*). Additionally, RECOMBINE identified recurrent composite markers that delineate the cellular hierarchy of each cell type (Fig. 4B; Supplemental Fig. S4C). The UMAP projection of cells based on RECOMBINE markers successfully separated all 23 cell types (Fig. 4C). Hierarchical clustering strength based on RECOMBINE markers was significantly higher compared with that based on an equal number of top DEGs (Fig. 4D). Although all RECOMBINE markers were included in the DEG set, the number of RECOMBINE markers (N = 366) represented only 12% of the total DEGs (N = 3096, absolute log_2_-fold-change >0.25 and adjusted *P* < 0.05), demonstrating optimized discriminative power with substantially fewer markers (Fig. 4E). Notably, RECOMBINE markers were enriched in highly significant DEGs but also included markers from less significant DEGs, suggesting that these less significant genes may play crucial roles in distinguishing hierarchical subpopulations (Supplemental Fig. S4B).

The hierarchical tissue structure and associated marker sets within the mouse cortex were decoded with a bottom to top analysis on the RECOMBINE output (Fig. 4B; Supplemental Fig. S4D). Starting from the bottom branches on the cell population tree, we computed the RECOMBINE features that are shared by the cells contained at each branch of the tree (Fig. 4B). Next, we moved up to a parental node to compute the shared features for the corresponding cell population. This was continued until reaching a cell hierarchy at which cell populations diverged and did not carry any shared RECOMBINE features. This approach enabled us to define hierarchical cell populations with lower hierarchies connected through shared features as well as higher hierarchies with no shared features. The application to the cortex tissue identified three distinct cell-type groups matched to excitatory, inhibitory, and nonneuronal compartments. Within each group, the connected and hierarchical cell identities shared common markers (Supplemental Fig. S4D). Although the cell types in each cluster were hierarchically linked to each other through the shared markers (Supplemental Fig. S4D), they were disconnected from the cell types in the other groups.

To spatially interrogate the mouse visual cortex, a panel of 1020 genes was selected and profiled in the STARmap data set (Wang et al. 2018). To compare the discriminative power of this panel with RECOMBINE-selected markers, we computed the silhouette score of cell-type labels based on the expression of each gene panel. RECOMBINE achieved nearly the same silhouette score as the STARmap panel while using far fewer genes (366 vs. 1020), indicating substantial redundancy in the original STARmap design (Supplemental Fig. S4E). A comparison between the 366 RECOMBINE markers and the 1020 STARmap genes revealed 248 overlapping markers (Fig. 4F). These 248 genes effectively discriminated all cell types in the scRNA-seq data set (Supplemental Fig. S4F), suggesting that they possessed sufficient power to resolve cell identities in spatial data. Using these genes, unsupervised clustering of the STARmap data identified 11 cell clusters across the excitatory neuron, inhibitory neuron, and nonneuronal compartments (Fig. 4G). Spatial mapping of these clusters recapitulated the layered organization of excitatory neurons and the sparse distribution of inhibitory neurons (Fig. 4H). The RECOMBINE markers formed distinct modules across cell clusters and defined cell subpopulations, whereas some markers were shared across clusters or compartments, reflecting the continuous nature of gene expression across cell types (Fig. 4I; Supplemental Fig. S4G). For example, *Slc6a1* and *Gad2* were enriched in inhibitory neurons but also expressed in astrocytes and oligodendrocytes, respectively. We hypothesized that the most enriched markers could form a reduced-size panel with minimal loss of discriminative power. To test this, we selected the top 10 enriched markers from each cluster, resulting in a panel of 69 markers. UMAP projections using these 69 markers effectively separated all cell subpopulations, closely mirroring the patterns observed with the full set of discriminant markers, except for cluster eL4 being farther from cluster eL5 (Supplemental Fig. S4H). These results demonstrate that RECOMBINE enables unbiased, data-driven selection of marker panels from paired scRNA-seq data, providing a robust foundation for targeted spatial transcriptomics.

### RECOMBINE defines CD8^+^ T cell states and markers predictive of cancer immunotherapy response

**Figure 5.**
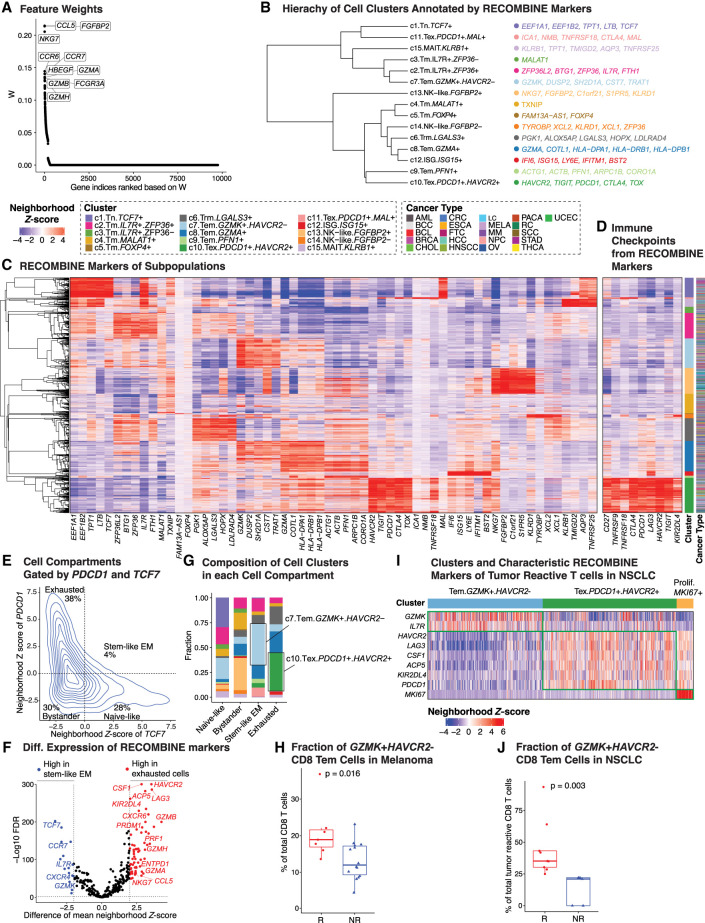
RECOMBINE identifies CD8^+^ T cell states and markers associated with cancer immunotherapy response. (*A*) Feature weights of all genes in which the top 10 discriminant markers are labeled. (*B*) Hierarchy of cell clusters annotated by top five RECOMBINE markers. (*C*) Heatmap of neighborhood *Z*-scores showing gene modules across clusters. (*D*) Heatmap of neighborhood *Z*-scores showing stimulatory and inhibitory immune checkpoint genes identified by RECOMBINE. (*E*) CD8^+^ T cell compartments gated by neighborhood *Z*-scores of *TCF7* and *PDCD1*. (EM) Effector memory. (*F*) Differentially expressed markers between stem-like EM and exhausted T cell compartments defined in *E*. (*G*) Compositions of cell clusters across the cell compartments defined in *E*. (*H*) Comparison of *GZMK*^+^*HAVCR2*^−^ Tem (c7 in RECOMBINE analysis) CD8^+^ T cell fractions in immunotherapy responders (R; N = 7) versus nonresponders (NR; N = 14) of melanoma patients treated with anti-PD-1 therapy (Sade-Feldman et al. 2018). (*I*) Heatmap of neighborhood *Z*-scores showing characteristic markers of clusters of 13,403 tumor-reactive CD8^+^ T cells from anti-PD-1-treated and treatment-naive patients with non-small-cell lung cancer (Liu et al. 2022). (*J*) Comparison of *GZMK^+^HAVCR2*^−^ Tem cell fractions within tumor-reactive CD8^+^ T cells in anti-PD-1 therapy responder (R; N = 9) versus nonresponder (NR; N = 5) patients with non-small-cell lung cancer.

Advancing cancer immunotherapies, such as immune checkpoint blockade and adoptive cell therapy, requires a comprehensive understanding of CD8^+^ T cell states and their associated markers (Van der Leun et al. 2020; Waldman et al. 2020; Chow et al. 2022; Lowery et al. 2022). Using RECOMBINE, we identified composite markers of CD8^+^ T cell states from a pancancer scRNA-seq data set comprising 234,550 CD8^+^ T cells (Zheng et al. 2021). RECOMBINE selected 320 discriminant markers that effectively distinguished subgroups of CD8^+^ T cells (Fig. 5A; Supplemental Fig. S5A; Supplemental Table S4). Among the top 10 ranked markers were cytotoxic molecules (e.g., *FGFBP2*, *FCGR3A*, *NKG7*, *GZMA*, *GZMB*, and *GZMH*) and cytokine-related and growth factor–related molecules (e.g., *CCL5*, *CCR6*, *CCR7*, and *FGFBP2*), reflecting effector and memory functions. Hierarchical clustering strength based on RECOMBINE markers was higher than that based on DEGs (absolute log_2_-fold-change >0.25 and adjusted *P* < 0.05) (Supplemental Fig. S5B). Unsupervised clustering of RECOMBINE markers revealed 15 clusters (Fig. 5B; Supplemental Fig. S5C), each characterized by composite markers that were either unique to specific clusters or shared among clusters with similar CD8^+^ T cell states (Fig. 5B,C). Examination of checkpoint gene expression highlighted cluster c10, which exhibited elevated levels of both stimulatory (e.g., *CD27*, *TNFRSF9*, *TNFRSF18*) and inhibitory (e.g., *CTLA4*, *PDCD1*, *LAG3*, *HAVCR2*, *TIGIT*) checkpoint molecules, suggesting a terminally exhausted state (Fig. 5D). Furthermore, cluster c10 showed enriched expression of *KIR2DL4*, an inhibitory checkpoint molecule typically expressed on NK cells, aligning with recent findings that an NK cell–like transition is a hallmark of CAR T cell dysfunction (Good et al. 2021). In summary, RECOMBINE accurately identified CD8^+^ T cell states and their characteristic markers, providing valuable insights for improving cancer immunotherapies.

We next investigated whether characteristic markers and CD8^+^ T cell subpopulations were associated with responses to immunotherapies, such as PD-1 blockade. Previous studies have indicated that exhausted CD8^+^ T cells (*TCF7*^−^*PDCD1*^+^) acquire a stable and distinct epigenetic profile that remains minimally remodeled after anti-PD-1 therapy (Pauken et al. 2016; Sen et al. 2016). In contrast, stem-like effector memory (EM) CD8^+^ T cells (*TCF7*^+^*PDCD1*^+^) provide tumor control and exhibit a proliferative burst in response to anti-PD-1 therapy (Im et al. 2016; Siddiqui et al. 2019; Huang et al. 2022). To identify characteristic markers and specific cell subpopulations responsive to anti-PD-1 therapy, we classified all CD8^+^ T cells into four compartments—naive-like, bystander, stem-like EM, and exhausted cells—based on neighborhood *Z*-scores of *TCF7* (a stemness marker) and *PDCD1* (an activation and exhaustion marker) (Fig. 5E). To distinguish stem-like EM cells from exhausted cells, we conducted differential analyses of the neighborhood *Z*-scores for discriminant markers between these two populations (Fig. 5F; Supplemental Table S4). As anticipated, stem-like EM cells were significantly enriched in naive and memory markers (e.g., *TCF7*, *CCR7*, *IL7R*, *CXCR4*), whereas exhausted cells were enriched in cytotoxic and terminal differentiation markers (e.g., *GZMA*, *GZMB*, *GZMH*, *PRF1*, *NKG7*, *CCL5*, *CXCR6*, *ENTPD1*, *PRDM1*). *GZMK* expression was significantly elevated in stem-like EM cells, suggesting a role in early-stage EM cell development, whereas inhibitory checkpoint molecules *HAVCR2*, *LAG3*, and *KIR2DL4* (but not *PDCD1*, *CTLA4*, or *TIGIT*) were highly enriched in exhausted cells, implicating these molecules in the late-stage development of terminally exhausted cells. In alignment with these findings, cluster c7 (*GZMK*^+^*HAVCR2*^−^, representing Tem cells) and cluster c10 (*PDCD1*^+^*HAVCR2*^+^, representing Tex cells) were identified as the largest subpopulations of stem-like EM and exhausted cells, respectively (Fig. 5G). These results highlight distinct markers and subpopulations that may serve as potential targets for improving anti-PD-1 immunotherapy efficacy.

As stem-like EM CD8^+^ T cells are likely the primary targets of anti-PD-1 therapy, we hypothesized that *GZMK*^+^*HAVCR*2^−^ Tem cells might play a pivotal role in mediating therapeutic responses. To test this, we analyzed the association of *GZMK*^+^*HAVCR2*^−^ Tem cells with clinical outcomes in a subset of anti-PD-1-treated melanoma patients (N = 21) described by Sade-Feldman et al. (2018). Consistent with our hypothesis, the enrichment of c7 (*GZMK*^+^*HAVCR2*^−^ Tem) cells was significantly higher in patients who responded to anti-PD-1 therapy (Fig. 5H). To validate this finding, we applied RECOMBINE to an independent scRNA-seq data set comprising 13,403 tumor-reactive CD8^+^ T cells from both anti-PD-1-treated and treatment-naive patients with non-small-cell lung cancer (NSCLC; N = 34) (Liu et al. 2022). Three major cell states were identified with characteristic markers: *GZMK*^+^*HAVCR2*^−^ Tem, *PDCD1*^+^*HAVCR2*^+^ Tex, and *MKI67*^+^ proliferative cells (Fig. 5I). Among posttherapy samples from this cohort (N = 14), the *GZMK*^+^*HAVCR2*^−^ Tem cell population was significantly enriched in responders, consistent with the findings in melanoma patients (Fig. 5J). Furthermore, the top markers of exhausted cells (e.g., *HAVCR2*, *LAG3*, *KIR2DL4*, *CSF1*, and *ACP5*) identified in the pancancer data set (Fig. 5F) were recapitulated in the NSCLC data set (Fig. 5I), reinforcing their roles in T cell exhaustion. Notably, *KIR2DL4*, *CSF1*, and *ACP5* remain less characterized in the context of T cell exhaustion, warranting further investigation to uncover their potential contributions. In conclusion, RECOMBINE analyses identified characteristic markers of stem-like EM and exhausted CD8^+^ T cells, demonstrated the enrichment of *GZMK*^+^*HAVCR2*^−^ Tem cells in responders to anti-PD-1 therapy, and proposed novel markers of T cell exhaustion. These findings provide a foundation for future studies aimed at improving cancer immunotherapy by targeting CD8^+^ T cell states and their associated markers.

### RECOMBINE identifies compact marker sets for diverse cell types across human tissues

**Figure 6.**
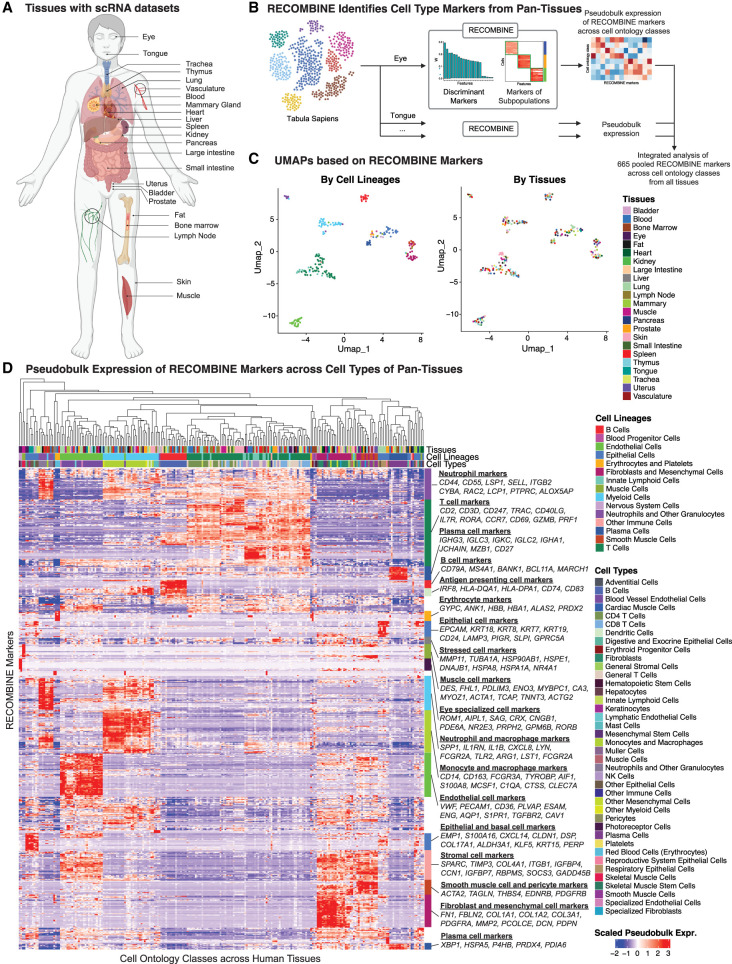
RECOMBINE identifies concise discriminant markers of cell types across human tissues. (*A*) Schematic of the tissues profiled by single-cell RNA sequencing from the Tabula Sapiens data set used in this study. (*B*) Workflow of RECOMBINE applied to the Tabula Sapiens to identify recurrent composite markers that effectively discriminate cell types in all tissues. (*C*) UMAP projections of pseudobulk expression of RECOMBINE markers across cell types in all tissues, colored by cell lineage (*left*) and tissue type (*right*). (*D*) Heatmap showing pseudobulk expression of RECOMBINE markers across cell types in all tissues.

Finally, we applied RECOMBINE to the Tabula Sapiens data set, a human single-cell transcriptomic reference atlas comprising nearly 500,000 cells from more than 20 tissues and organs (Tabula Sapiens Consortium et al. 2022). This analysis aimed to evaluate RECOMBINE's capacity to identify discriminant markers across heterogeneous cell contexts in a large-scale data set while generating a comprehensive resource of tissue marker sets. Single-cell transcriptomic data and cell ontology class annotations were collected for tissues from the Tabula Sapiens (Fig. 6A). For each tissue, RECOMBINE identified recurrent composite marker genes for cell ontology classes (Fig. 6B; Supplemental Table S5). To enable integrated analysis, we averaged expression data to generate pseudobulk expression matrices, which were then pooled across all tissues. UMAP projections based on RECOMBINE markers revealed that transcriptomic variation was primarily driven by cell lineages rather than tissue of origin (Fig. 6C). Hierarchical clustering further demonstrated RECOMBINE's ability to identify selective markers unique to specific cell types and shared functional markers spanning multiple lineages (Fig. 6D). For example, well-known markers such as *CD79A*, *MS4A1*, and *BANK1* were identified for B cells, whereas immunoglobulin genes (*IGHG3*, *IGLC3*, *IGKC*) were linked to plasma cells. Skeletal muscle cells exhibited selective expression of genes like *ENO3*, *MYBPC1*, *CA3*, *MYOZ1*, and *ACTA1*, whereas eye-specific cells, such as photoreceptors and Müller cells, uniquely expressed *ROM1*, *AIPL1*, *SAG*, *CRX*, and *CNGB1*. Additionally, shared markers included genes related to antigen presentation (*HLA-DQA1*, *HLA-DPA1*, *CD74*) found in B cells, macrophages, and dendritic cells, as well as stress-related heat shock protein genes (*HSP90AB1*, *HSPE1*, *HSPA1A*) spanning multiple lineages. In conclusion, RECOMBINE effectively identified both specific and shared markers across heterogeneous cell contexts, underscoring its utility for comprehensive human pantissue transcriptomic analyses.

## Discussion

Here, we present the development and applications of RECOMBINE, a computational framework designed to select optimized and composite markers that discriminate hierarchical cell identities. RECOMBINE is built on the sparse hierarchical clustering with the SHC-SSL algorithm, which outperformed the conventional sparse hierarchical clustering with LASSO (SHC) in both optimal marker selection and accurate clustering, as demonstrated in our simulation studies. Evaluations across diverse biological data sets revealed that RECOMBINE markers exhibited high robustness to hyperparameter variation and data sparsity and outperformed other feature selection methods including both DEG- and non-DEG-based methods. RECOMBINE couples marker selection with hierarchical clustering. This coupling allows RECOMBINE to identify composite markers that discriminate both cell types and dynamic states with high granularity. The simultaneous optimization of marker selection and hierarchical clustering enables RECOMBINE to detect markers across all levels of the cell and tissue hierarchy, including distinct cell types, continuous cell states, and rare cell subpopulations. Despite its simplicity, this unique capability underscores its utility for advancing our understanding of cellular heterogeneity in complex biological systems.

We demonstrated RECOMBINE's versatility through its application across diverse biological domains. Using a scRNA-seq data set of zebrafish embryogenesis and a Slide-seq data set of the mouse cerebellum, RECOMBINE characterized transcriptional continuums associated with developmental time and spatial gradients. Using scRNA-seq data from the mouse visual cortex, RECOMBINE identified discriminant markers for cell types and states. These markers successfully delineated spatially resolved cell subpopulations in the same tissue using a STARmap data set, highlighting RECOMBINE's robust performance in marker selection for targeted spatial transcriptomics. In scRNA-seq data from pancancer CD8^+^ T cells, RECOMBINE identified composite markers for CD8^+^ T cell states, including *GZMK*^+^*HAVCR2*^*−*^ EM cells associated with anti-PD-1 therapy response, and novel markers related to CD8^+^ T cell exhaustion. Finally, RECOMBINE identified composite marker sets for heterogeneous cell types across more than 20 human tissues in the Tabula Sapiens. Furthermore, application of RECOMBINE to a mouse intestinal scRNA-seq data set enabled the identification of selective markers defining a rare Reg4^+^ enteroendocrine cell subpopulation (Supplemental Material). In scRNA-seq data sets of malignant cells from breast cancer and melanoma, RECOMBINE uncovered expression programs underlying both inter- and intratumoral heterogeneity (Supplemental Material). Taken together, RECOMBINE demonstrates broad applicability for data-driven extraction of recurrent composite marker sets to characterize diverse cell subpopulations across biological systems.

One key application of RECOMBINE is the selection of concise gene panels for targeted spatial or single-cell assays. SpaPros was developed to address this task by combining DEG-based and PCA loading–based feature selection algorithms (Kuemmerle et al. 2024). However, both approaches underperformed relative to RECOMBINE in our benchmarking analyses. Furthermore, DEGs and PCA loadings are independent, decoupled procedures, whereas RECOMBINE integrates discriminant marker selection directly with hierarchical clustering, capturing both discrete and continuous cell states within a unified framework. Thus, RECOMBINE provides a more effective strategy for gene panel selection compared with DEG- and PCA-based methods.

When the number of genes in a targeted assay is limited, RECOMBINE supports two complementary strategies for marker selection. First, if the goal is to capture continuous cell-state variation, feature weights from RECOMBINE can be used as importance scores to select the top-ranked genes. Based on our systematic benchmarking, the top markers identified by RECOMBINE or its fixed-parameter variant, fRECOMBINE, are highly robust to hyperparameter variation and data sparsity, and capture the largest proportion of total gene-expression variance compared with other methods. Second, if the objective is to recover discrete cell types, the top recurrent composite markers from each cell subpopulation can be pooled to construct the final gene panel. As demonstrated in the STARmap data set of the mouse visual cortex, a reduced panel comprising the top 10 genes per cluster (ranked by mean neighborhood *Z*-score and fraction of significant cells) effectively separated all cell types.

RECOMBINE introduces several key algorithmic advances over existing methods. First, RECOMBINE does not require a prespecified marker panel size; instead, it determines an optimal size through a statistically principled and data-driven approach using the gap statistic. In contrast, methods like SCMER (Liang et al. 2021) require the user to define the marker panel size a priori, which is often unknown. This adaptive approach ensures that RECOMBINE selects marker panels tailored to the underlying data. Second, RECOMBINE enforces marker sparsity using a SHC-SSL penalty function, which has demonstrated superior marker selection performance compared with the LASSO penalty function employed in methods like SHC, scGeneFit (Dumitrascu et al. 2021), and RANKCORR (Vargo and Gilbert 2020). Third, RECOMBINE incorporates neighborhood enrichment tests to identify marker modules associated with cell subpopulations, enabling biologically meaningful interpretations of cell types and states. Finally, a notable advantage of RECOMBINE is its ability to select an optimized and compact set of discriminant markers. Compared with DEGs, RECOMBINE produces a substantially smaller marker set while maintaining high sensitivity. This compactness facilitates easier interpretation and practical utility of the marker list but may result in some loss of comprehensiveness. As observed in our benchmark study, SHC-SSL achieved a median precision of 100% but a median recall of 79%. This imperfect recall can be attributed to the fact that some markers may be omitted if the selected markers already provide maximum discrimination for cell subpopulations.

RECOMBINE is a scalable algorithm capable of analyzing hundreds of thousands of cells, as demonstrated in the pancancer CD8^+^ T cell analysis with 234,550 cells. Achieving this scalability required addressing the computational challenge inherent to RECOMBINE's marker selection algorithm, SHC-SSL, which operates on a dissimilarity matrix of dimension *n*^2^ by *p*, (*n*: cells; *p*: features), leading to quadratic increases in memory usage as *n* grows. To overcome this limitation, we implemented a pseudocell strategy that merges cells with shared transcriptomic profiles into pseudocells and identifies markers that discriminate between these pseudocells. Provided the pseudocells represent fine-grained cell groups, this approach approximates the solution for selecting discriminant markers at the single-cell level. An additional benefit of the pseudocell strategy is its ability to reduce technical noise and stabilize variance, thereby enabling more robust marker selection, as demonstrated in our benchmarking analyses. Notably, similar strategies have been employed by methods such as MetaCell and SEACells, which construct granular and distinct cell group representations to address sparsity in single-cell data while preserving fine-grained heterogeneity of cell states. The outputs from MetaCell and SEACells, representing granular cell groups, can also be seamlessly integrated into RECOMBINE as an alternative to the pseudocell strategy, serving as inputs for marker selection. With these strategies, we anticipate that RECOMBINE will remain highly scalable for applications in large-scale projects (Rood et al. 2022).

RECOMBINE is a generalized framework for the unbiased selection of recurrent composite markers to characterize hierarchical biological identities. Its applications, as demonstrated through diverse examples, can uncover how multicellular systems are organized into hierarchical cell identities that mediate biological functions. By generating marker modules for cell subpopulations, RECOMBINE can identify drivers of key biological processes. For instance, it can detect immune cell-state-specific marker sets for subpopulations implicated in resistance to immunotherapy. Moreover, RECOMBINE markers can inform the development of new assays for identifying unique processes, cell types, and dynamic states within tissues using single-cell omic data. Although our studies focused primarily on single-cell expression profiles, RECOMBINE is adaptable to a variety of omic data modalities, including protein expression, epigenomic profiles, and genomic alterations, in both single-cell and bulk resolutions. This versatility positions RECOMBINE as a valuable tool for diverse biological analyses. Through its capacity to discover novel drivers of biological functions, characterize rare cell subpopulations in complex tissues, and guide strategies for manipulating biological processes (e.g., selection of therapeutic interventions), RECOMBINE has the potential to profoundly advance our understanding of biological systems and enhance our ability to manipulate their functions.

## Methods

### Overview of RECOMBINE

Given single-cell omic data, RECOMBINE uncovers recurrent composite marker sets of hierarchical cell identities in two steps. In the first step, RECOMBINE employs the SHC-SSL algorithm to select the markers that best discriminate cells while performing hierarchical clustering. The discriminant markers consist of a minimal set that characterizes the data and can be subjected to overrepresentation analysis for biological interpretation. Besides hierarchical clustering, the cell dissimilarity matrix based on the discriminant markers can be used for hierarchical or partitional clustering. In the second step, RECOMBINE uses neighborhood recurrence tests to extract markers of cell subpopulations from the discriminant markers. Specifically, the cell dissimilarity matrix is used to build a *k*-nearest neighbors graph of cells. For any individual cell, its nearest neighbors compose its local neighborhood context. Then, a neighborhood *Z*-score is calculated to quantify the recurrence of each of the discriminant markers for each cell's local neighborhood. Finally, based on statistical significances of neighborhood *Z*-scores, markers are identified at the single-cell level; based on fractions of cells having significant neighborhood *Z*-scores, markers are identified at the subpopulation level.

Algorithmic details are provided in the Supplemental Methods. We begin by describing the SHC-based algorithms, including a review of the previously developed SHC method (Witten and Tibshirani 2010), which we applied to identify recurrent oncogenic coalterations (Li et al. 2022), and the introduction of two related algorithms, SHC-SSL and SHC-FL. SHC-SSL incorporates a spike-and-slab LASSO penalty (Ročková 2018; Ročková and George 2018), whereas SHC-FL employs a fused LASSO penalty (Tibshirani et al. 2005). Because these approaches involve hyperparameters, we next present a strategy for hyperparameter selection based on gap statistics (Tibshirani et al. 2001). We then describe the simulation data sets and benchmarking metrics used to evaluate the three algorithms. As SHC-SSL can be computationally intensive for large sample sizes, we further introduce a pseudocell strategy to reduce computational cost. Finally, we present a neighborhood recurrence test to extract both cell-level and subpopulation-level markers from discriminant features. The RECOMBINE package is implemented in R (R Core Team 2023), and all analyses were performed in R unless otherwise specified.

In the following sections, we detail the evaluation of the method and its application to diverse biological data sets.

### Evaluation of RECOMBINE's robustness to hyperparameter variation and data sparsity

We evaluated the robustness of RECOMBINE to hyperparameter variation and data sparsity using a single-cell RNA-seq data set of murine hematopoiesis (Dahlin et al. 2018). To assess sensitivity to hyperparameter settings, λ_1_ was fixed at 0.0001, and λ_0_ was varied between 0.001 and 4000. For each λ_0_, RECOMBINE generated a set of feature weights and top-ranked features matched in size to the optimal λ_0_ feature set.

To evaluate robustness under different sparsity conditions, the scRNA-seq data were randomly downsampled with probabilities (P) ranging from 0.1 to 0.9 at three levels: transcript, cell, and gene. At the transcript level, each UMI was treated as an independent event, and nonzero counts were sampled from a binomial distribution with probability P. At the cell level, cells were uniformly sampled without replacement with probability P. At the gene level, genes were uniformly sampled without replacement with probability P. All downsampled data sets were preprocessed identically to the full data set and analyzed using RECOMBINE.

Performance stability was assessed using Pearson's and Spearman's correlations between feature weights derived from each λ_0_ (or downsampled data) and those obtained at the optimal λ_0_ (or the complete data). In addition, robustness of the top 50 features was quantified using a robustness score, as described previously (Li et al. 2022). This score measures the overlap between the top N^*^ features, here N^*^ = 50, from the ranked feature list F* and the reference feature list *F*, weighted by feature rank in the reference:

$$\mathrm{Robustness}\;\mathrm{Score}=\frac{\sum_{i=1}^{N^{*}}\sum_{\;j=1}^{N}w_{j}I(F_{\left(i\right)}^{*}=F_{\left(j\right)})}{N^{*}},$$

where *I*( · ) is an indicator function, and *w*_*j*_ represents a rank scaled weight of feature *j* for 1 ≤ *j* ≤ *N*. The rank-scaling is formulated as

$$w_{j}=\{\begin{array}{cc} \; & \;1,j\leq N^{*},\;\\ \frac{2N^{*}-j}{N^{*}},\; & \;N^{*}<j\leq 2N^{*},\;\\ \;\; & 0,\;\mathrm{otherwise}.\; \end{array}$$

where the top-ranked N* features contribute most strongly to the robustness score, and lower-ranked features have progressively smaller contributions in proportion to their ranks.

### Benchmarking of RECOMBINE and other feature selection methods using biological data sets

We benchmarked the performance of RECOMBINE against other feature selection methods, including its fixed hyperparameter variant fRECOMBINE (λ_1_ = 0.0001, λ_0_ = 1). Six additional methods were evaluated for comparison: three DEG-based approaches (Wilcoxon test, negative binomial test, and OVR-LR) and three non-DEG-based approaches (HVGs, Hotspot, and PCA loadings). For each data set, RECOMBINE was used to select an optimized set of discriminant markers, whereas other methods were constrained to select the same number of features as the RECOMBINE marker set.

#### Data collection

Four biological data sets were used for benchmarking. For murine hematopoiesis (Dahlin et al. 2018), raw count matrices for six wild-type samples from Lin^−^ Sca1^+^ Kit^+^ (LSK) and Lin^−^ Kit^+^ (LK) sorting gates were downloaded from https://www.ncbi.nlm.nih.gov/geo/query/acc.cgi?acc=GSE107727. For murine hematopoiesis (Nestorowa et al. 2016), log_2_-transformed counts and cell population annotations were obtained from https://blood.stemcells.cam.ac.uk/single_cell_atlas. For murine hematopoiesis (Paul et al. 2015), raw count data and annotations (paul15_clusters) were retrieved using the SCANPY (Wolf et al. 2018) function sc.datasets.paul15(). For *C. elegans* embryogenesis (Packer et al. 2019), expression data and developmental annotations were downloaded from https://depts.washington.edu/trapnell-lab/software/monocle3/celegans/data/.

#### Data preprocessing

Low-quality cells were filtered following the preprocessing criteria described in the original publications. The remaining cells were log-normalized. Batch correction across samples or batches was applied using Harmony for the data sets of Dahlin et al. (2018) and Packer et al. (2019). Cell-type annotations were obtained from the original studies when available. For Dahlin et al. (2018), cell labels were reassigned to 19 clusters derived by unsupervised clustering using all genes.

#### RECOMBINE and fRECOMBINE

To construct pseudocells (miniclusters), the Seurat (Hao et al. 2021) function FindClusters was applied with a resolution of 50. Pseudocell expression profiles were defined as the average expression of cells within each minicluster. RECOMBINE was applied to the pseudocell expression profiles to select an optimized set of discriminant features. fRECOMBINE was applied to the same pseudocell data using fixed hyperparameters to select the top-weighted features matching the size of the RECOMBINE marker set.

#### DEG-based methods

DEG-based methods used FindClusters in Seurat for clustering, followed by FindAllMarkers to identify markers for each cluster in a one-versus-rest manner. Three statistical tests were evaluated: Wilcoxon rank-sum test, negative binomial test, and logistic regression. Genes with a log_2_-fold-change >0.25 and adjusted *P* < 0.05 were retained. Within each cluster, genes were ranked by adjusted *P-*value (ties broken by descending log_2_-fold-change). A combined DEG list was generated by evenly selecting top-ranked markers from each cluster such that the total number of selected genes matched the RECOMBINE marker count.

#### Highly variable genes

HVGs were identified using the Seurat function FindVariableFeatures with the variance-stabilizing transformation method. Features were ranked by standardized variance, and the top features were selected to match the size of the RECOMBINE marker set.

#### Hotspot

Following Hotspot preprocessing guidelines, log_2_-normalized data were scaled, and PCA was performed with 10 components using SCANPY. A Hotspot object was created with n_neighbors = 30 to compute a neighborhood graph, and autocorrelation scores were calculated using compute_autocorrelations(). Genes with FDR < 0.05 were retained. The top features ranked by Hotspot *Z*-scores were selected to match the RECOMBINE marker set size.

#### PCA loadings

For PCA loadings, the log_2_-normalized expression matrix was centered (but not scaled) per gene. The Seurat function RunPCA was used to compute feature loadings. Each feature was scored by the sum of the absolute values of its loadings across all PCs, and the top features were selected to match the RECOMBINE marker set size.

#### Evaluation metrics

Performance was assessed using three quantitative metrics: fraction of variance explained (FVE), which measures how well selected features capture total transcriptomic variance, canonical correlation (CC), which measures similarity between embeddings derived from selected versus all features, and gap statistic, which measures the hierarchical clustering strength of selected features relative to random features.

To compute FVE, the centered (but unscaled) single-cell expression matrix was decomposed by PCA using the top 10 PCs as the data embedding representing total variance. A multivariate regression was then performed to regress these PCs on the expression matrix of selected features; the ratio of fitted to total variance yielded the FVE.

For CC, PCA was also performed on the selected features, and the top 10 PCs were used as the embedding. Canonical correlation analysis (cancor in R) was conducted between the two embeddings, and the mean correlation across all dimensions was used as the final CC score.

The gap statistic was defined as

$$\mathrm{Gap}=\log O(X)-\frac{1}{N}\overset{N}{\underset{i=1}{\sum }}\log O(X_{i}),$$

where **X** represents the expression matrix of the selected markers, **X**_*i*_ denotes the expression matrix of a random gene set of the same size as **X**, and the objective function **X** is defined according to the sparse hierarchical clustering framework described earlier. Gap statistics were computed using pseudocell expression matrices for all methods after feature selection. For DEG-based methods, gene weights were uniformly assigned and normalized to an L2 norm of one. For non-DEG-based methods, feature importance scores were normalized to the same L2 constraint.

### Applying RECOMBINE to biological data sets for in-depth case studies

We applied RECOMBINE to bulk and single-cell omic data sets. In each analysis, we set λ_1_ = 0.0001 and chose the optimal λ_0_ based on gap statistic profile. The squared distance was used as the dissimilarity metric in SHC-SSL. The dissimilarity matrix based on discriminant markers was subjected to building a *k*-nearest neighbors graph, where K = 20, followed by Leiden clustering, in which the choice of resolution parameter depends on the data sets. The cells were visualized in heatmaps showing Leiden clusters, in which cells within each Leiden cluster were hierarchically clustered using the average linkage.

#### Identifying discriminant markers characterizing continuous cell-state transitions during zebrafish development

We analyzed a single-cell RNA-seq data set of zebrafish embryogenesis (Farrell et al. 2018). The Seurat object (URD_Zebrafish_Object.rds) and corresponding metadata (URD_Dropseq_Meta.txt) were downloaded from the Single-Cell Portal of the Broad Institute (https://singlecell.broadinstitute.org/single_cell/study/SCP162). The expression count matrix was extracted from the Seurat object. Cells with fewer than three expressed genes and genes expressed in fewer than 200 cells were excluded. Expression data were log_2_-normalized, and 2000 HVGs were selected for dimensionality reduction using the top 20 PCs. The resulting embeddings were used to construct shared nearest neighbors graphs, and unsupervised clustering with a resolution of 30 generated 289 pseudocells. RECOMBINE was applied to the pseudocell data to identify discriminant markers, which were then mapped back to the full scRNA-seq data set to extract recurrent composite markers across all cells.

#### Selecting discriminant markers of transcriptional variation shaped by spatial gradients in the mouse cerebellum

A Slide-seq data set of the mouse cerebellum (Puck_180819_12) was downloaded from the Single-Cell Portal of the Broad Institute (https://singlecell.broadinstitute.org/single_cell/study/SCP354). Bead barcodes with fewer than 50 detected genes and genes expressed in fewer than 20 beads were excluded, and beads lacking spatial coordinate information were removed. Expression data were log_2_-normalized, and 2000 HVGs were selected for dimensionality reduction using the top 10 PCs based on the elbow plot. The resulting embeddings were used to construct shared nearest neighbors graphs, and unsupervised clustering with a resolution of 15 produced 220 pseudocells. RECOMBINE was then applied to the pseudocell data to identify discriminant markers. Because the gap statistic profile plateaued with increasing λ_0_, the optimal λ_0_ was chosen according to the one–standard error rule, defined as one standard error below the maximum gap statistic value. To compute Moran's *I*, spatial autocorrelation analysis was performed using the R package spdep. A *k*-nearest neighbors graph (K = 8) of spatial locations was constructed using knearneigh(). Neighbor lists were converted into spatial weights with nb2listw(), and Moran's *I* was calculated using moran.test() with a two-sided alternative hypothesis.

#### Selecting targeted panel from scRNA data for spatial molecular profiling of mouse visual cortex

For scRNA data, we downloaded an scRNA-seq data set of mouse visual cortex from Allen Brain Atlas (Tasic et al. 2018; http://celltypes.brain-map.org/api/v2/well_known_file_download/694413985) and filtered out cells with low quality. In total, we obtained 14,249 cells of 23 annotated cell types belonging to glutamatergic neurons, GABAergic neurons, and nonneuronal cells. We employed Seurat to normalize the data using the “LogNormalize” method and selected 10,000 HVGs using the “vst” method. Before applying RECOMBINE to the scRNA data, to reduce computational cost of SHC-SSL imposed by the large numbers of cells, we generated 287 pseudocells corresponding to clusters identified by Leiden clustering with a resolution of 50. Then, we used RECOMINE to select discriminant markers from 10,000 HVGs of these pseudocells. As a result, 366 genes were selected as discriminant markers of the mouse visual cortex scRNA data.

For spatial data, we downloaded a STARmap data set of 1020 genes mapped in mouse primary visual cortex (Wang et al. 2018; https://figshare.com/articles/dataset/STARmap_datasets/22565209). The spatial location coordinate of each cell was extracted from “labels.npz” according to the method provided by the original paper describing STARmap (Wang et al. 2018). In total, there were 1549 cells. We employed Seurat to normalize the data using the “LogNormalize” method with a scale factor of 100. After clustering, we annotated cell clusters into cell types based on expression of marker genes. Specifically, *Slc17a7* and *Gad1* were used to annotate excitatory and inhibitory neurons, respectively; *Nov*, *Rorb*, *Sulf2*, and *Pcp4* were used to annotate eL2/3, eL4, eL5, and eL6 neurons, respectively; *Vip*, *Sst*, and *Pvalb* were used to annotate VIP, SST, and PVALB neurons, respectively; and *Pdgfra*, *Aqp4*, *Enpp2*, and *Bsg* were used to annotate microglia, astrocyte, oligodendrocyte, and endothelial cells, respectively. As the paired scRNA data of mouse visual cortex did not include microglia cells, we filtered out microglia cells from the spatial data, resulting in 1486 spatially resolved cells remaining in our analysis. Of 366 discriminant markers identified from the scRNA data, 248 genes were mapped in the spatial data of mouse visual cortex. Next, we performed Leiden clustering of 1486 cells from the spatial data using the 248 discriminant markers, for which a resolution of 0.5 was used. Cells were annotated based on their markers and mapped to their physical locations. Finally, we performed RECOMBINE neighborhood recurrence tests to identify markers that characterize cell subpopulations in the spatial data.

#### Identifying markers that characterize cell states of CD8^+^ tumor-infiltrating T cells across cancer types

We downloaded the SingleCellExperiment object of scRNA-seq data set of pancancer CD8^+^ T cells (https://zenodo.org/record/5461803), which was obtained by integrating 234,550 tumor-infiltrating CD8^+^ T cells of 316 patients across 21 cancer types from multiple sources (Zheng et al. 2021). To reduce noise, cells within each source had been partitioned into small groups (“miniclusters”). In total, the expression matrix had a dimension of 11,972 miniclusters by 11,772 genes. The downloaded data had been log_2_-transformed, and 1500 HVGs were provided. Before applying RECOMBINE to the data set, we generated pseudocells using Seurat to reduce computational cost of SHC-SSL. Specifically, the 1500 HVGs were scaled and subjected to PCA, from which the top 50 principal components were used for embedding. The embeddings were corrected using Harmony to mitigate batch effects across data sources. Then, shared nearest neighbors graphs were constructed, and Leiden clustering with a resolution of 30 was used to generate 205 pseudocells. We applied RECOMBINE to the pseudocell data to select discriminant markers and extract markers from the discriminant markers for all cells. In Leiden clustering of all cells based on the selected discriminant markers, the resolution parameter was set to two, which identified 15 clusters, including naive cells expressing *TCF7* (c1); memory cells expressing *IL7R*, *MALAT1*, or *FOXP4* (c2–c5); resident memory cells expressing *LGALS3* (c6); EM cells expressing *GZMK*, *GZMA*, or *PFN1*, (c7–c9); exhausted cells simultaneously expressing high levels of *PDCD1*, *CTLA4*, *TIGIT*, and *TOX* (c10–c11); interferon-stimulated gene (ISG)–positive cells (c12); natural killer (NK)–like cells expressing both *NKG7* and *KLRD1* (c13–c14); and mucosal-associated invariant T cells (MAIT) expressing both *KLRB1* and *TMIGD2* (c15).

To study compositions of cell clusters associated with anti-PD-1 therapy response, we downloaded the clinical response data from the report by Sade-Feldman et al. (2018; Supplemental Table 1, sheet patient-scRNA data) and used data of patients treated with anti-PD-1 therapy, in which responders (N = 7) included patients with complete and partial responses and nonresponders (N = 14) included those with stable or progressive disease based on RECIST. The pancancer data set included 3853 CD8^+^ T cells from these 21 patients. The statistical comparison of cell compositions between responders and nonresponders was based on Wilcoxon rank-sum test.

To validate the association between the composition of *GZMK*^+^*HAVCR2*^−^ EM T cells and anti-PD-1 response, we used a data set of CD8^+^ T cells from NSCLC patients (N = 34) treated with anti-PD-1 therapy in the report by Liu et al. (2022), which was not included in the pancancer CD8^+^ T cell data set. We downloaded scRNA-seq, scTCR-seq, and metadata from the NCBI Gene Expression Omnibus (GEO; https://www.ncbi.nlm.nih.gov/geo/; GSE179994) and response data from Liu et al. (2022; Supplemental Table 1). Of 34 patients, 11 patients had posttreatment samples (N = 14) with annotated clinical responses (nine responders vs. five nonresponders). Based on the scTCR-seq and metadata, we extracted all CD8^+^ T cells having TCR clonotypes shared by exhausted CD8^+^ T cells, resulting in 13,403 cells defined as tumor-reactive CD8^+^ T cells. After constructing 286 pseudocells, we applied RECOMBINE to this data set and selected 236 discriminant genes. Leiden clustering of all tumor-reactive CD8^+^ T cells with the discriminant genes identified three clusters: *GZMK*^+^*HAVCR2*^−^ EM, exhausted, and proliferative T cells. Finally, we compared compositions of *GZMK*^+^*HAVCR2*^−^ EM T cells from posttreatment samples between responders and nonresponders after anti-PD-1 therapy.

#### Identifying markers that characterize a rare subpopulation of mouse intestine

We downloaded an scRNA-seq data set of intestinal organoid cells from the GEO (GSE62270). The data were preprocessed as described previously (Grün et al. 2015). Mitochondrial genes, ERCC spike-ins, and genes associated with clustering artifacts (*Rn45s*, *Malat1*, *Kcnq1ot1*, *A630089N07Rik*, and *Gm17821*) were excluded as described previously (Grün et al. 2015; Gehart et al. 2019). The resulting data set had an expression matrix of 238 cells by 3773 genes. Finally, log_2_ transformation was applied. Using this preprocessed data set, we ran RECOMBINE to identify markers that discriminate both common and rare cell subpopulations. In Leiden clustering, the resolution parameter was set to 0.6. RECOMBINE identified a *Reg4*^+^*Tac1*^+^ enteroendocrine cell population, which was further validated using PanSci, a single-cell atlas of mouse tissues (Zhang et al. 2025). The PanSci UCSC Cell Browser (https://cells.ucsc.edu/?ds=mouse-pansci) was used to visualize UMAP embeddings showing cell distributions and the expression of *Reg4* and *Tac1* across the duodenum, ileum, and jejunum.

#### Selecting markers that characterize intertumoral heterogeneity in malignant cells from melanoma patients

From the GEO (GSE72056) (Tirosh et al. 2016), we downloaded an scRNA-seq data set that had been integrated from 14 melanoma patients and log_2_-transformed. Following the method described by Tirosh et al. (2016), we processed the data by filtering out cells with either fewer than 1700 detected genes or mean housekeeping expression below three. We kept only malignant cells in our analysis. Furthermore, we removed ERCC spike-ins and excluded genes with a pooled expression below four. The resulting data had 1252 cells by 22,568 genes. Before applying RECOMBINE, we generated pseudocells using Seurat. Next, 2000 HVGs were selected, scaled, and subjected to PCA, from which the top 10 principal components were used for embedding and clustering. Then, Leiden clustering with a resolution of 20 was used to generate 106 pseudocells. We used RECOMBINE to select discriminant markers that characterize the heterogeneity of these pseudocells. In Leiden clustering of all cells based on the selected discriminant markers, the resolution parameter was set to 0.3.

To calculate MYC and immune signature scores of patients from TCGA, we downloaded the TCGA melanoma RNA-seq V2 expression data set including 443 patients (Akbani et al. 2015) and log_2_-transformed the RSEM-based gene quantifications to calculate signature scores. We also downloaded the corresponding clinical data for survival analysis. According to MYC signature scores, patients were classified as either MYC^lo^ (less than the median) or MYC^hi^ (greater than the median), and according to immune signatures, patients were classified as either Immune^lo^ (less than the median) or Immune^hi^ (greater than the median). Together, we stratified patients into four groups (MYC^lo^ Immune^lo^, MYC^lo^ Immune^hi^, MYC^hi^ Immune^lo^, and MYC^hi^ Immune^hi^) and compared their survival times.

#### Selecting markers that characterize transcriptional intratumoral heterogeneity of TNBC

We downloaded scRNA-seq data sets of two TNBC patients, denoted as TNBC1 and TNBC2, from the GEO (GSE148673) reported by Gao et al. (2021). In these data sets, cells had been annotated as either tumor or normal cells. To study the heterogeneity of tumor cells, we removed the normal cells, resulting in 797 cells in TNBC1 and 620 cells in TNBC2. Next, 2000 HVGs were selected, scaled, and subjected to PCA, from which the top 10 principal components were used for embedding and clustering. Leiden clustering with a resolution of 10 was used to generate 48 and 56 pseudocells for TNBC1 and TNBC2, respectively. Then, we ran RECOMBINE to select discriminant markers that characterize transcriptional intratumoral heterogeneity in each TNBC individual. In Leiden clustering of all the cells based on the selected discriminant markers, the resolution parameters were set to 0.3 for both TNBC1 and TNBC2.

#### Identifying markers that discriminate heterogeneous cells of human tissues in Tabula Sapiens

We downloaded h5ad files containing raw counts and cell ontology class annotations from the resource website (https://tabula-sapiens.sf.czbiohub.org/) of the Tabula Sapiens Consortium et al. (2022). Data preprocessing and RECOMBINE were applied to each tissue individually before integrating the results for combined analysis. For each tissue, cells with fewer than 200 expressed genes, cells in the top 10th percentile of expressed gene counts, and cells with >15% of transcripts originating from mitochondrial genes were excluded. The data were then log-normalized. PCA was performed, selecting 30 principal components based on elbow plots, and batch effects across donors were corrected using Harmony. Miniclusters were identified via unsupervised clustering using the Seurat FindClusters function with a resolution of 30, and pseudocell expression profiles were calculated as the average expression of cells within each minicluster. RECOMBINE was applied to the pseudocell expression data to select discriminant markers, followed by neighborhood recurrence tests to compute neighborhood *Z*-scores and adjusted *P*-values. RECOMBINE markers were extracted for each cell ontology class by selecting the top 10 markers with >50% of cells showing significant neighborhood recurrence and an average neighborhood *Z*-score greater than two. Subsequently, the expression profiles of cells within the same ontology class were averaged to construct a pseudobulk expression matrix of cell ontology classes for each tissue. These pseudobulk matrices were pooled to generate an integrated expression matrix of cell ontology classes across all tissues based on RECOMBINE markers. Finally, UMAP projection and hierarchical clustering were performed to visualize the relationships among cell ontology classes and to uncover patterns of RECOMBINE markers across diverse cell types.

### Code availability

The RECOMBINE R package and simulation data are available at GitHub (https://github.com/korkutlab/recombine) and as Supplemental Code.

## Supplemental Material

Supplement 1

Supplement 2

Supplement 3

Supplement 4

Supplement 5

Supplement 6

Supplement 7

Supplement 8

Supplement 9

Supplement 10
