# Supplementary material for: RECOMBINE identifies recurrent composite markers of cell types and states
Source: Genome Res. 2026 Jun;36(6):1221–37. doi: 10.1101/gr.280817.125 (PMC13262949; doi:10.1101/gr.280817.125)
Supplement: Supplement 1 [file Supplemental_Methods.docx]

**Supplemental Methods**

Here, we describe some notations. For any vector $\boldsymbol{v\in}\mathbb{R}^{n}$, $\left\| \mathbf{v} \right\|$ denotes the L2 norm (Euclidian norm) of $\mathbf{v}$, and $\left\| \mathbf{v} \right\|_{1}$ denotes the L1 norm of $\mathbf{v}$.

**Sparse hierarchical clustering**

First, we summarize the previously developed SHC algorithm (Witten and Tibshirani, 2010). Given an $n\times p$ data matrix $\mathbf{X}$ with $n$ samples by $p$ features, we denote $x_{i}$ as a sample that is a $p$-dimensional vector indexed by$i$, where $1\leq i\leq n$ and $d\left( x_{i},x_{i^{'}} \right)$ as a measure of the dissimilarity between samples $x_{i}$ and $x_{i^{'}}$. Assuming the dissimilarity measure is additive in features, that is, $d\left( x_{i},x_{i^{'}} \right)=\sum_{j} d_{i,i^{'},j}$, where $d_{i,i^{'},j}$ is the dissimilarity measure between samples $x_{i}$ and $x_{i^{'}}$ along feature $j$, the dissimilarity matrix becomes the solution of a constrained optimization problem:

$$\mathbf{U}^{\mathbf{*}}=\underset{\mathbf{U}}{\mathrm{argmax}} \left\{ \sum_{j} \sum_{i,i^{'}} d_{i,i^{'},j}U_{i,i^{'}} \right\} subject to \sum_{i,i^{'}} U_{i,i^{'}}^{2}\leq1.$$

It can be shown as the optimal solution $U_{i,i^{'}}^{*}\propto\sum_{j} d_{i,i^{'},j}$. Thus, with this reformulation, hierarchical clustering performed on $\mathbf{U}^{\mathbf{*}}$ is equivalent to that performed on $\left\{ d\left( x_{i},x_{i^{'}} \right) \right\}_{n\times n}$. To calculate $d_{i,i^{'},j}$, one could use the squared Euclidean distance $d_{i,i^{'},j}=\left( X_{ij}-X_{i^{'}j} \right)^{2}$ or the absolute difference $d_{i,i^{'},j}=\left| X_{ij}-X_{i^{'}j} \right|$.

The weighted dissimilarity matrix was introduced in the SHC method. SHC provides a means to select a small number of discriminant features that contribute to a weighted dissimilarity matrix. Specifically, SHC solves the optimization problem

$$\underset{\mathbf{w,U}}{\mathrm{maximize}} \left\{ \sum_{j} w_{j}\sum_{i,i^{'}} d_{i,i^{'},j}U_{i,i^{'}} \right\},$$

$$subject to \sum_{i,i^{'}} U_{i,i^{'}}^{2}\leq1, \left\| \mathbf{w} \right\|^{2}\leq1, \left\| \mathbf{w} \right\|_{1}\leq s, w_{j}\geq0 \forall j,$$

where $\mathbf{w}$ is a weight vector for features and $s$ is a tuning parameter that controls sparsity of $\mathbf{w}$. When $s$ is small, the LASSO, or L1, penalty of $\mathbf{w}$ encourages many elements of$\mathbf{w}$ to be zero. The L2 penalty of $\mathbf{w}$ is necessary because without it, only one element of $\mathbf{w}$ would be nonzero. Let $\mathbf{U}^{\mathbf{**}}$ optimize the above criterion, from which we have $U_{i,i^{'}}^{**}\propto\sum_{j} w_{j}d_{i,i^{'},j}$, where the dissimilarity along each feature is weighted according to $w_{j}$. Due to these constraints, a small value of $s$ makes $w$ sparse, so $\mathbf{U}^{\mathbf{**}}$ depends only on a small subset of features. Therefore, clustering based on $\mathbf{U}^{\mathbf{**}}$ depends only on the selected features.

To simplify representation, let’s rearrange $\left\{ d_{i,i^{'},j} \right\}_{n\times n\times p}$ to get a matrix $\mathbf{D}\in\mathbb{R}^{n^{2}\times p}$ in which column $j$ consists of ordered elements $\left\{ d_{i,i^{'},j} \right\}_{n\times n}$ stacked into a vector. Accordingly, let $\mathbf{u}\in\mathbb{R}^{n^{2}}$ be the vector by stacking $\left\{ U_{i,i^{'}} \right\}_{n\times n}$. Then, the SHC optimization criterion is in the form

$$\underset{\mathbf{w,u}}{\mathrm{minimize}} \left\{ -\mathbf{u}^{T}\mathbf{Dw} \right\},$$

$$subject to \left\| \mathbf{u} \right\|^{2}\leq1, \left\| \mathbf{w} \right\|^{2}\leq1, \left\| \mathbf{w} \right\|_{1}\leq s, w_{j}\geq0 \forall j.$$

We can iteratively optimize $\mathbf{u}$ and $\mathbf{w}$. With $\mathbf{w}$ fixed, this criterion is equivalent to

$$\underset{\mathbf{u}}{\mathrm{minimize}} \left\{ -\mathbf{u}^{T}\mathbf{Dw} \right\} subject to \left\| \mathbf{u} \right\|^{2}\leq1.$$

The optimal $\mathbf{u=}\frac{\mathbf{Dw}}{\left\| \mathbf{Dw} \right\|}$, and $u_{i}\geq0$ for $1\leq i\leq n^{2}$ since all the elements of $\mathbf{D}$ and $\mathbf{w}$ are nonnegative. With $\mathbf{u}$ fixed, the criterion takes the form

$$\underset{\mathbf{w}}{\mathrm{minimize}} \left\{ -\mathbf{u}^{T}\mathbf{Dw} \right\} subject to \left\| \mathbf{w} \right\|^{2}\leq1, \left\| \mathbf{w} \right\|_{1}\leq s,$$

where we drop the non-negativity constraint because it is redundant when all the elements of $\mathbf{u}$ and $\mathbf{D}$ are non-negative. The optimal $\mathbf{w}=\frac{S\left( \mathbf{D}^{T}\mathbf{u,}\Delta\right)}{\left\| S\left( \mathbf{D}^{T}\mathbf{u,}\Delta\right) \right\|}$, where $S$ is the soft-thresholding operator $S\left( x,c \right)\boldsymbol{=}\mathrm{sign}\left( x \right)\left( \left| x \right|-c \right)_{+}$, and $\Delta=0$ if this results in $\left\| \mathbf{w} \right\|_{1}\leq s$; otherwise, $\Delta>0$ is chosen such that $\left\| \mathbf{w} \right\|_{1}=s$.

**Sparse hierarchical clustering with spike-and-slab LASSO**

Here, we introduce the SHC-SSL algorithm, which uses an SSL penalty. We start with a spike-and-slab Laplace prior distribution of $\mathbf{w}$, from which an SSL penalty function follows (Ročková and George, 2018; Ročková, 2018). Suppose the prior distribution of $\mathbf{w}$ is in the form

$$\pi\left( \mathbf{w} | \gamma\right)= \prod_{j=1}^{p} \left[ \left( 1-\gamma_{j} \right)\psi_{0}\left( w_{j} \right)+\gamma_{j}\psi_{1}\left( w_{j} \right) \right],$$

$$\psi_{0}\left( w \right)= \frac{\lambda_{0}}{2}e^{-\lambda_{0}\left| w \right|},$$

$$\psi_{1}\left( w \right)= \frac{\lambda_{1}}{2}e^{-\lambda_{1}\left| w \right|},$$

where $\boldsymbol{\gamma}\mathbf{:=}\left( \gamma_{j} \right)_{1\leq j\leq n}$, $\gamma_{j}\in\left\{ 0, 1 \right\}$. Each $w_{j}$ is a mixture of two Laplace distributions, $\psi_{0}$ and $\psi_{1}$, and $\gamma_{j}$ is the latent variable indicating the mixture components to which $w_{j}$ belongs. With $\lambda_{0}$ large, $\psi_{0}\left( w \right)$ serves a restrictive, point-mass-like “spike distribution” for modeling a small $w$; in contrast, with $\lambda_{1}$ small, $\psi_{1}\left( w \right)$ serves a diffusive, heavy-tailed “slab distribution” for modeling a large $w$. Suppose the latent variable $\boldsymbol{\gamma}$ has a hierarchical prior distribution as

$$\pi\left( \boldsymbol{\gamma} | \theta\right)= \prod_{j=1}^{p} \theta^{\gamma_{j}}\left( 1-\theta\right)^{1-\gamma_{j}},$$

$$\pi\left( \theta\right)= \mathrm{Beta}\left( a,b \right),$$

where $\theta$ is shared across $\gamma_{j}$’s and $\mathrm{Beta}\left( a,b \right)$ is the beta distribution with shape parameters $a,b>0$. As we can see, $\pi\left( \gamma_{j}=1 | \theta\right)=\theta$; thus, $\theta$ acts as the prior probability of $w_{j}$ being large. Since $\theta$ is shared across $w_{j}$’s, intuitively $\theta$ reflects the expected fraction of large $w_{j}$’s. By marginalizing out $\boldsymbol{\gamma}$, we obtain

$$\pi\left( \mathbf{w} | \theta\right)= \prod_{j=1}^{p} \left[ \left( 1-\theta\right)\psi_{0}\left( w_{j} \right)+\theta\psi_{1}\left( w_{j} \right) \right].$$

Furthermore, by marginalizing out $\theta$, we have

$$\pi\left( \mathbf{w} \right)= \int_{0}^{1} \prod_{j=1}^{p} \left[ \left( 1-\theta\right)\psi_{0}\left( w_{j} \right)+\theta\psi_{1}\left( w_{j} \right) \right]d\pi\left( \theta\right).$$

With this prior marginal distribution of $\mathbf{w}$, we define the SSL penalty as

$$\mathrm{pen}_{SSL}\left( \mathbf{w} \right)= -\log\frac{\pi\left( \mathbf{w} \right)}{\pi\left( \mathbf{0}_{\boldsymbol{p}} \right)},$$

which is centered so that $\mathrm{pen}_{SSL}\left( \mathbf{0} \right)=0$. Note that our definition of the SSL penalty is the negative of the one described by Ročková and George (2018). We choose this definition because it is consistent with the definition of the LASSO penalty that is the negative logarithm of the Laplace distribution. When $\lambda_{0}\to\infty$ and $\lambda_{1}\to0$, the SSL penalty is equivalent to an L0-norm penalty; when $\lambda_{0}=\lambda_{1}$, it is equivalent to a LASSO penalty. Therefore, the SSL penalty forms a continuous bridge between an L0-norm and a LASSO penalty. We keep $\lambda_{1}$ to a small constant and tune $\lambda_{0}$ during hyperparameter selection.

Now, we replace the LASSO with the SSL penalty in the objective of SHC, yielding the optimization criterion as

$$\underset{\mathbf{w,u}}{\mathrm{minimize}} \left\{ \mathbf{-u}^{T}\mathbf{Dw+}\mathrm{pen}_{SSL}\left( \mathbf{w} \right) \right\},$$

$$subject to \left\| \mathbf{u} \right\|^{2}\leq1, \left\| \mathbf{w} \right\|^{2}\leq1, w_{j}\geq0 \forall j.$$

We iteratively optimize $\mathbf{u}$ and $\mathbf{w}$. With $\mathbf{w}$ fixed, the optimal $\mathbf{u}$ is calculated in the same way as the one in SHC. With $\mathbf{u}$ fixed, the SHC-SSL objective takes the form

$$\underset{\mathbf{w}}{\mathrm{mimimize}} \left\{ -\mathbf{u}^{T}\mathbf{Dw+}\mathrm{pen}_{SSL}\left( \mathbf{w} \right) \right\} subject to \left\| \mathbf{w} \right\|^{2}\leq1.$$

To find stationary points of the above objective, we rewrite it as a Lagrangian function

$$\mathcal{L}\left( \mathbf{w}\boldsymbol{,}\alpha\right)=-\mathbf{u}^{T}\mathbf{Dw+}\frac{1}{2}\alpha\left\| \mathbf{w} \right\|^{2}+\mathrm{pen}_{SSL}\left( \mathbf{w} \right),$$

where $\alpha\geq0$ is the Lagrange multiplier and a factor of $1/2$ is included for later convenience. Following the sub-differential calculus, we obtain

$$\frac{\partial\mathcal{L}\left( \mathbf{w}\boldsymbol{,}\alpha\right)}{\partial w_{j}}=-y_{i}\mathbf{+}\alpha w_{j}+\frac{\partial\mathrm{pen}_{SSL}\left( \mathbf{w} \right)}{\partial\left| w_{j} \right|}h\left( w_{j} \right),$$

where $\mathbf{y}=\mathbf{D}^{T}\mathbf{u}$ and $h\left( w \right)$ is a subgradient of $\left| w \right|$, meaning $h\left( w \right)=\mathrm{sign}\left( w \right)$ when $w\neq0$, and $h\left( w \right)\in\left[ -1, 1 \right]$ when $w=0$.

Let’s present the derivative of the SSL penalty. Using Lemma 2 and 3 described by Ročková and George (2018), the derivative of the SSL penalty with respect to $\left| w_{j} \right|$ is in the form

$$\frac{\partial\mathrm{pen}_{SSL}\left( \mathbf{w} \right)}{\partial\left| w_{j} \right|}= \lambda^{*}\left( w_{j}; \mathbf{w}_{\backslash j} \right),$$

where

$$\lambda^{*}\left( w_{j}; \mathbf{w}_{\backslash j} \right)= \left( 1-p^{*}\left( w_{j}; \mathbf{w}_{\backslash j} \right) \right)\lambda_{0}+p^{*}\left( w_{j}; \mathbf{w}_{\backslash j} \right)\lambda_{1},$$

$$p^{*}\left( w_{j}; \mathbf{w}_{\backslash j} \right)=\frac{\theta_{j}\psi_{1}\left( w_{j} \right)}{\left( 1-\theta_{j} \right)\psi_{0}\left( w_{j} \right)+\theta_{j}\psi_{1}\left( w_{j} \right)},$$

$$\theta_{j}=E\left[ \theta| \mathbf{w}_{\backslash j} \right],$$

where $E\left[ \theta| \mathbf{w}_{\backslash j} \right]$ is the posterior expectation of $\theta$ given $\mathbf{w}_{\backslash j}$. Here, $\lambda^{*}\left( w_{j}; \mathbf{w}_{\backslash j} \right)$ can be interpreted as a weighted average of $\lambda_{0}$ and $\lambda_{1}$, and the weight $p^{*}\left( w_{j}; \mathbf{w}_{\backslash j} \right)$ is the conditional probability of $w_{j}$ being from $\psi_{1}\left( w_{j} \right)$ rather than from $\psi_{0}\left( w_{j} \right)$. Thus, a larger $w_{j}$ tends to have a smaller $\lambda^{*}\left( w_{j}; \mathbf{w}_{\backslash j} \right)$. Both $\lambda^{*}\left( w_{j}; \mathbf{w}_{\backslash j} \right)$ and $p^{*}\left( w_{j}; \mathbf{w}_{\backslash j} \right)$ depend on $\theta_{j}$. To calculate $\theta_{j}=E\left[ \theta| \mathbf{w}_{\backslash j} \right]$, note that when $p$ is large, $E\left[ \theta| \mathbf{w}_{\backslash j} \right]$ should be similar to $E\left[ \theta| \mathbf{w} \right]$. From Lemma 4 in Ročková and George (2018), $\theta_{j}$ can be approximated by

$$\theta_{j}\approx E\left[ \theta| \mathbf{w} \right]\approx\frac{a+\left\| \mathbf{w} \right\|_{0}}{a+b+p},$$

where $\left\| \mathbf{w} \right\|_{0}$ is the L0 norm of $\mathbf{w}$.

Now, we solve for the optimal $\mathbf{w}$. Substituting the derivative of $\mathrm{pen}_{SSL}\left( \mathbf{w} \right)$, the derivative of $\mathcal{L}\left( \mathbf{w}\boldsymbol{,}\alpha\right)$ can be expressed as

$$\frac{\partial\mathcal{L}\left( \mathbf{w}\boldsymbol{,}\alpha\right)}{\partial w_{j}}=-y_{i}\mathbf{+}\alpha w_{j}+\lambda^{*}\left( w_{j}; \mathbf{w}_{\backslash j} \right)h\left( w_{j} \right).$$

Let $\hat{\mathbf{w}}$ denote an estimate of the global optimum of $\mathbf{w}$. At stationary points, with the Karush-Kuhn-Tucker conditions, $\hat{\mathbf{w}}$ needs to satisfy

$$y_{i}-\alpha\hat{w}_{j}\mathbf{=}\lambda^{*}\left( \hat{w}_{j}; {\hat{\mathbf{w}}}_{\backslash j} \right)\mathrm{sign}\left( \hat{w}_{j} \right) \mathrm{for} w_{j}\neq0,$$

$$\left| y_{i}-\alpha\hat{w}_{j} \right|\boldsymbol{\leq}\lambda^{*}\left( \hat{w}_{j}; {\hat{\mathbf{w}}}_{\backslash j} \right) \mathrm{for} w_{j}=0.$$

This can be written equivalently as

$$\hat{w}_{j}=\frac{1}{\alpha}S\left( y_{i},\lambda^{*}\left( \hat{w}_{j}; {\hat{\mathbf{w}}}_{\backslash j} \right) \right).$$

where the soft thresholding operator $S\left( x,c \right)\boldsymbol{=}\mathrm{sign}\left( x \right)\left( \left| x \right|-c \right)_{+}$. At optimum, $\alpha$ is chosen such that $\left\| \hat{\mathbf{w}} \right\|^{2}=1$.

It should be noted that the above necessary conditions are not sufficient for the global mode under the SSL penalty (Ročková and George, 2018). Using Theorem 3.1 from Ročková (2018), the global mode $\hat{\mathbf{w}}$ under the SSL penalty can be expressed as

$$\hat{w}_{j}=\frac{1}{\alpha}\tilde{S}\left( y_{i},\lambda^{*}\left( \hat{w}_{j}; {\hat{\mathbf{w}}}_{\backslash j} \right),\Delta\right),$$

$$\Delta\approx\sqrt{-2\log\left[ p^{*}\left( 0; {\hat{\mathbf{w}}}_{\backslash j} \right) \right]}+\lambda_{1},$$

where $\tilde{S}$ is a generalized thresholding operator defined as $\tilde{S}\left( x,c,\Delta\right)\boldsymbol{=}\mathrm{sign}\left( x \right)\left( \left| x \right|-c \right)_{+}\mathbb{I}\left( \left| x \right|>\Delta\right)$, and $\alpha$ is chosen such that $\left\| \hat{\mathbf{w}} \right\|^{2}=1$. We can think of $\tilde{S}$as a composition operator consisting of an L0-type hard thresholding operator $S_{0}\left( x,\Delta\right)\boldsymbol{=}x\mathbb{I}\left( \left| x \right|>\Delta\right)$ followed by a LASSO-type soft thresholding operator $S_{1}\left( x,c \right)\boldsymbol{=}\mathrm{sign}\left( x \right)\left( \left| x \right|-c \right)_{+}$. Both operators adaptively depend on the value of $\hat{\mathbf{w}}$. Each coordinate of $\hat{\mathbf{w}}$ has its own self-adaptive shrinkage, which borrows information across other coordinates. The hard thresholding operator $S_{0}$ depends on $\Delta$, which in turn depends on $p^{*}\left( 0; {\hat{\mathbf{w}}}_{\backslash j} \right)$, the conditional probability of $\hat{w}_{j}$ being zero is from the slab distribution. A larger $p^{*}\left( 0; {\hat{\mathbf{w}}}_{\backslash j} \right)$, corresponding to a smaller $\Delta$, induces a smaller hard thresholding effect on $\hat{w}_{j}$ (i.e., $S_{0}$ tends to leave $\hat{w}_{j}$ as is). In contrast, a larger hard thresholding effect is induced if $\hat{w}_{j}$ being zero is likely from the spike distribution (i.e., $S_{0}$ tends to set $\hat{w}_{j}$ as 0). On the other hand, the soft thresholding operator $S_{1}$ adaptively depends on $\lambda^{*}\left( \hat{w}_{j}; {\hat{\mathbf{w}}}_{\backslash j} \right)$. As we discussed before, a larger $\hat{w}_{j}$ has a smaller $\lambda^{*}\left( \hat{w}_{j}; {\hat{\mathbf{w}}}_{\backslash j} \right)$. When $\hat{w}_{j}$ is large, it is shrunk by a small amount (close to $\lambda_{1}$); when $\hat{w}_{j}$ is small, it is shrunk by a large amount (close to $\lambda_{0}$). Therefore, in contrast to the constant shrinkage of the LASSO penalty, the SSL penalty induces a self-adaptive shrinkage that depends on itself and borrows information from other coordinates. In other words, while LASSO constantly biases $\hat{w}_{j}$ toward zero (Hastie et al. 2015), SSL shrinks strongly small $\hat{w}_{j}$’s toward zeros but only introduces very slight biases for large $\hat{w}_{j}$’s. For example, as shown in Fig. 1c, by fixing $\lambda_{1}=0.001$ and increasing $\lambda_{0}$ from 1 to 50, the SSL penalty forms a continuum between a LASSO and an L0-norm penalty. When $\lambda_{0}=1$, the SSL penalty function is hardly distinguishable from LASSO. When $\lambda_{0}=50$, the SSL penalty function has a sharp transition from zero to non-zero weights of features, approaching the L0-norm penalty that is known to be ideal for feature selection.

SHC-SSL algorithm

Input: stacked dissimilarity matrix $\mathbf{D}$, shape parameters $a$ and $b$, and hyperparameter $\lambda_{0}$ and $\lambda_{1}$, where $\lambda_{1}$ is a small number and $\lambda_{0}>\lambda_{1}$.

Output: feature weights $\mathbf{w}$, dissimilarity matrix $\mathbf{U}$.

(1) Initialize $\mathbf{w}$ as $w_{1}=\cdots=w_{p}=\frac{1}{\sqrt{p}}.$

(2) Iterate until convergence of $\mathbf{w}$:

(a) Update $\mathbf{u}=\frac{\mathbf{Dw}}{\left\| \mathbf{Dw} \right\|}$.

(b) Update $\mathbf{w}$:

(i) Calculate $\theta=\frac{a+\left\| \mathbf{w} \right\|_{0}}{a+b+p}$.

(ii) Calculate $\mathbf{p}^{\mathbf{*}}=\frac{\theta\psi_{1}\left( \mathbf{w} \right)}{\left( 1-\theta\right)\psi_{0}\left( \mathbf{w} \right)+\theta\psi_{1}\left( \mathbf{w} \right)}$, where $\psi_{0}\left( w_{j} \right)= \frac{\lambda_{0}}{2}e^{-\lambda_{0}\left| w_{j} \right|}$ and $\psi_{1}\left( w_{j} \right)= \frac{\lambda_{1}}{2}e^{-\lambda_{1}\left| w_{j} \right|}$ for $1\leq j\leq p$.

(iii) Calculate $\boldsymbol{\lambda}^{\mathbf{*}}= \left( \mathbf{1}-\mathbf{p}^{\mathbf{*}} \right)\lambda_{0}+\mathbf{p}^{\mathbf{*}}\lambda_{1}$.

(iv) Calculate $\boldsymbol{\Delta}=\sqrt{-2\log\left[ \mathbf{p}^{\mathbf{*}} \right]}+\lambda_{1}$.

(v) Update $\mathbf{w}=\frac{\tilde{S}\left( {\mathbf{D}^{\boldsymbol{'}}}^{T}\mathbf{u,}\boldsymbol{\lambda}^{\mathbf{*}}\boldsymbol{, \Delta} \right)}{\left\| \tilde{S}\left( {\mathbf{D}^{\boldsymbol{'}}}^{T}\mathbf{u,}\boldsymbol{\lambda}^{\mathbf{*}}\boldsymbol{, \Delta} \right) \right\|}$ where $\tilde{S}\left( x,c,\Delta\right)\boldsymbol{=}\mathrm{sign}\left( x \right)\left( \left| x \right|-c \right)_{+}\mathbb{I}\left( \left| x \right|>\Delta\right)$.

(3) Calculate dissimilarity matrix:

(a) Update $\mathbf{u}=\frac{\mathbf{Dw}}{\left\| \mathbf{Dw} \right\|}$.

(b) Rewrite $\mathbf{u}$ as a $n\times n$ matrix $\mathbf{U}$.

(4) Perform clustering or dimension reduction using $\mathbf{U}$.

**Sparse hierarchical clustering with fused LASSO**

Here, we introduce the SHC-FL algorithm. An FL penalty contains L1 norms of both features and their successive differences (Tibshirani et al. 2005), which is defined as

$$\mathrm{pen}_{FL}\left( \mathbf{w} \right)= \lambda_{1}\sum_{j=1}^{p} \left| w_{j} \right|+\lambda_{2}\sum_{j=2}^{p} \left| w_{j}-w_{j-1} \right|,$$

where $\lambda_{1},\lambda_{2}\geq0$ are hyperparameters. While $\lambda_{1}$ encourages sparsity of features, $\lambda_{2}$ encourages neighboring features to be similar and some to be identical.

We replace the LASSO with the FL penalty in the objective of SHC, yielding the optimization criterion as

$$\underset{\mathbf{w,u}}{\mathrm{minimize}} \left\{ \mathbf{-u}^{T}\mathbf{Dw}+\mathrm{pen}_{FL}\left( \mathbf{w} \right) \right\},$$

$$subject to \left\| \mathbf{u} \right\|^{2}\leq1, \left\| \mathbf{w} \right\|^{2}\leq1, w_{j}\geq0 \forall j.$$

We iteratively optimize $\mathbf{u}$ and $\mathbf{w}$. With $\mathbf{w}$ fixed, the optimal $\mathbf{u}$ is calculated in the same way as the one in SHC. With $\mathbf{u}$ fixed, the SHC-FL objective takes the form

$$\underset{\mathbf{w}}{\mathrm{minimize}} \left\{ \mathbf{-u}^{T}\mathbf{Dw}+\mathrm{pen}_{FL}\left( \mathbf{w} \right) \right\} subject to \left\| \mathbf{w} \right\|^{2}\leq1,$$

which is convex, but not smooth and not separable. To find stationary points of the above objective, we rewrite it as a Lagrangian function

$$\mathcal{L}\left( \mathbf{w}\boldsymbol{,}\alpha\right)=-\mathbf{u}^{T}\mathbf{Dw+}\frac{1}{2}\alpha\left\| \mathbf{w} \right\|^{2}+\mathrm{pen}_{FL}\left( \mathbf{w} \right),$$

where $\alpha\geq0$ is the Lagrange multiplier. The derivative of $\mathcal{L}\left( \mathbf{w}\boldsymbol{,}\alpha\right)$ can be expressed as

$$\frac{\partial\mathcal{L}\left( \mathbf{w}\boldsymbol{,}\alpha\right)}{\partial w_{j}}=-y_{j}\mathbf{+}\alpha w_{j}+\frac{\partial\mathrm{pen}_{FL}\left( \mathbf{w} \right)}{\partial w_{j}},$$

where $\mathbf{y}=\mathbf{D}^{T}\mathbf{u}$. At stationary points, the optimal $w_{j}$’s needs to satisfy

$$-y_{j}\mathbf{+}\alpha w_{j}+\frac{\partial\mathrm{pen}_{FL}\left( \mathbf{w} \right)}{\partial w_{j}}=0.$$

Let’s present the derivative of the FL penalty. Following the sub-differential calculus, the derivative of $\mathrm{pen}_{FL}\left( \mathbf{w} \right)$ is in the form

$$\frac{\partial\mathrm{pen}_{FL}\left( \mathbf{w} \right)}{\partial w_{j}}=\left\{ \begin{aligned} \lambda_{1}h\left( w_{1} \right)-\lambda_{2}h\left( w_{2}-w_{1} \right), &j=1 \\ \lambda_{1}h\left( w_{j} \right)+\lambda_{2}\left[ h\left( w_{j}-w_{j-1} \right)-h\left( w_{j+1}-w_{j} \right) \right], &1<j\leq p, \end{aligned} \right.$$

where $h\left( x \right)$ is a subgradient of $\left| x \right|$, meaning $h\left( x \right)=\mathrm{sign}\left( x \right)$ when $x\neq0$ and $h\left( x \right)\in\left[ -1, 1 \right]$ when $x=0$. It is easy to see ${\partial\mathrm{pen}_{FL}\left( \alpha\mathbf{w} \right)}/{\partial\left( \alpha w_{j} \right)}={\partial\mathrm{pen}_{FL}\left( \mathbf{w} \right)}/{\partial w_{j}}$, where $\alpha$ is a positive constant. Thus, a scaling of $\mathbf{w}$ does not affect the derivative of $\mathrm{pen}_{FL}\left( \mathbf{w} \right)$.

Now, we solve for the optimal $\mathbf{w}$. Let $\mathbf{w}^{\boldsymbol{'}}\boldsymbol{=}\alpha\mathbf{w}$. Because ${\partial\mathrm{pen}_{FL}\left( \mathbf{w}^{\boldsymbol{'}} \right)}/{\partial w_{j}^{'}}={\partial\mathrm{pen}_{FL}\left( \mathbf{w} \right)}/{\partial w_{j}}$, the necessary condition of the stationary points of $\mathbf{w}$ becomes

$$-y_{j}\mathbf{+}w_{j}^{'}+\frac{\partial\mathrm{pen}_{FL}\left( \mathbf{w}^{\boldsymbol{'}} \right)}{\partial w_{j}^{'}}=0.$$

To solve $\mathbf{w}^{\boldsymbol{'}}$ in the above equation, we construct an axillary convex optimization problem whose solution coincides with $\mathbf{w}^{\boldsymbol{'}}$:

$$\underset{\mathbf{a}}{\mathrm{minimize}} \left\{ \frac{1}{2}\left\| \mathbf{a}\boldsymbol{-y} \right\|^{2}+\mathrm{pen}_{FL}\left( \mathbf{a} \right) \right\},$$

where $\mathbf{a}\in\mathbb{R}^{p}$. This is a signal approximation problem under the fused LASSO penalty, with the solution called the fused LASSO signal approximator (FLSA). We employ an FLSA solver proposed by Liu et al. (2010), because of its high computational efficiency given a pair of $\lambda_{1}$ and $\lambda_{2},$and strong scalability in high dimension (Xin et al. 2014). Once $\mathbf{w}^{\boldsymbol{'}}$ is obtained, we get $\mathbf{w=}{\mathbf{w}^{\boldsymbol{'}}}/\left\| \mathbf{w}^{\boldsymbol{'}} \right\|$ such that $\left\| \mathbf{w} \right\|^{2}=1$.

SHC-FL algorithm

The algorithm is same as the algorithm of SHC-SSL, with the following differences:

Input: stacked dissimilarity matrix $\mathbf{D}$ in which features are ordered, outlier indicator $\mathbf{z}$, and hyperparameter ${\lambda_{1},\lambda}_{2}\geq0$.

During the iteration, step 2b is replaced with:

(2b) Update $\mathbf{w}$:

(i) Find $\mathbf{w}^{\boldsymbol{'}}=\underset{\mathbf{a}}{\mathrm{argmin}} \left\{ \frac{1}{2}\left\| \mathbf{a}\boldsymbol{-}{\mathbf{D}^{\boldsymbol{'}}}^{T}\mathbf{u} \right\|^{2}+\lambda_{1}\sum_{j=1}^{p} \left| a_{j} \right|+\lambda_{2}\sum_{j=2}^{p} \left| a_{j}-a_{j-1} \right| \right\}$ using an FLSA solver.

(ii) Update $\mathbf{w}=\frac{\mathbf{w}^{\boldsymbol{'}}}{\left\| \mathbf{w}^{\boldsymbol{'}} \right\|}$.

**Hyperparameter selection**

We now turn to the selection of hyperparameters. In the above algorithms, the hyperparameters include: (1) $s$ in SHC; (2) $\left( \lambda_{1},\lambda_{2} \right)$ in SHC-FL; and (3) $\lambda_{0}$ in SHC-SSL, where we keep $\lambda_{1}$ to a small constant. Let $\boldsymbol{\lambda}$ denote a single or a pair of hyperparameters in any one of the above algorithms. As in SHC, we employ a permutation-based approach, the gap statistic, to select the optimal value of $\boldsymbol{\lambda}$ (Witten and Tibshirani, 2010; Tibshirani et al. 2001). Given a hyperparameter $\boldsymbol{\lambda}$, the gap statistic measures the strength of the clustering based on real data with respect to the one based on randomly permuted data that are supposed to have no cluster. We quantify the strength of the clustering as

$$O\left( \boldsymbol{\lambda},\mathbf{X} \right)=\sum_{j} w_{j}\sum_{i,i^{'}} d_{i,i^{'},j}U_{i,i^{'}},$$

where $\mathbf{X}$ denotes the dataset used. Let $\mathbf{X}$ be the original real dataset, and $\mathbf{X}_{1},\ldots,\mathbf{X}_{B}$ denote $B$ randomly permuted datasets by permuting samples within each feature. The gap statistic is calculated by

$$\mathrm{Gap}\left( \boldsymbol{\lambda} \right)=\log O\left( \boldsymbol{\lambda},\mathbf{X} \right)-\frac{1}{B}\sum_{b=1}^{B} \log O\left( \boldsymbol{\lambda},\mathbf{X}_{b} \right).$$

The optimal value of $\boldsymbol{\lambda}$ is obtained at the largest gap statistic.

In addition, to accelerate convergence of the presented algorithms for a series of $N$ hyperparameter candidates $\left\{ \boldsymbol{\lambda}_{1},\ldots,\boldsymbol{\lambda}_{N} \right\}$, we employ a “warm start” strategy: since solutions for similar hyperparameters are close, $\mathbf{w}$ is initialized as a solution for a nearby hyperparameter so that its solution can be found relatively quickly.

**Pseudo-cell strategy for reducing computational cost of SHC-SSL**

All SHC-based algorithms involve a dissimilarity matrix $\mathbf{D}\in\mathbb{R}^{n^{2}\times p}$, making the requirements of memory and computation scale quadratically with the sample size $n$. Thus, it is expensive to naïvely apply SHC-SSL when $n$ is large. To reduce the computational cost, one can group cells with similar transcriptomes to form pseudo-cells, which are then subjected to SHC-SSL to select discriminant features. The selected features are used in the final clustering of all cells. An additional advantage of the pseudo-cell strategy is that it reduces technical noise and stabilizes variance, which may lead to more robust feature selection.

To construct pseudo-cells, specifically, one can use any efficient clustering algorithm, such as the Leiden algorithm, to group cells into many small clusters. Then, within each small cluster, pseudo-cells can be formed by calculating the mean of expressions of its constituent cells. This strategy is similar to the one used in Vision (DeTomaso et al. 2019), where pseudo-cells were termed “micro-clusters”. We employed this strategy for datasets with a large number of cells to reduce computational cost and select discriminant markers that best discriminate pseudo-cells.

**Neighborhood recurrence test**

To test if a marker of a cell is recurrently up- or downregulated in a biological subgroup rather than randomly perturbed, we devised a statistical test based on the idea that a recurrent marker should be enriched in the local neighborhood where the cell is located. The neighborhood recurrence test consists of two steps. First, we construct a K nearest neighbor graph of cells using their similarities based on discriminant markers. We define $S_{k}\left( i \right)$ to be the local neighborhood of cell $i$, for $1\leq i\leq n$, where $S_{k}\left( i \right)$ includes the $k$-nearest neighbors of $i$. Second, we perform a statistical test to determine if marker $j$ is significantly upregulated or downregulated in the local neighborhood $S_{k}\left( i \right)$. Let $\mu_{j}$ and $\sigma_{j}^{2}$denote the mean and variance of the expression of marker $j$, which can be estimated as $\mu_{j}= {\sum_{i} X_{ij}}/n$ and $\sigma_{j}^{2}= {\sum_{i} \left( X_{ij}-\mu_{j} \right)^{2}}/\left( n-1 \right),$ respectively. The mean expression of marker $j$ for cells in the local neighborhood $S_{k}\left( i \right)$ can estimated as

$$\bar{X}_{S_{k}\left( i \right),j}= \frac{\sum_{i^{'}\in S_{k}\left( i \right)} X_{i^{'}j}}{k}.$$

By the central limit theorem, when $k$ is sufficiently large, $\bar{X}_{S_{k}\left( i \right),j}$ will be approximately normal distributed with its mean as $\mu_{j}$ and variance as $\sigma_{j}^{2}/k$. In our studies, we chose $k=20$. Next, we calculate the neighborhood *Z* score of marker $j$ for cell $i$ as

$$Z_{ij}= \frac{\bar{X}_{S_{k}\left( i \right),j}-\mu_{j}}{\sigma_{j}/\sqrt{k}}.$$

Thus, the distribution of the neighborhood *Z* score is approximately the standard normal distribution, i.e., $Pr\left( Z \right)=N(0, 1)$. Accordingly, the *P*-value of marker $j$ that is recurrently up- or downregulated in the local neighborhood of a cell $i$ is $Pr\left( Z>Z_{ij} \right)$ or $Pr\left( Z<Z_{ij} \right)$, respectively. Finally, we adjust the *P*-values of all tested markers for each cell using the Benjamini-Hochberg procedure.

**Simulation data**

We generated simulation datasets by adapting a simulation procedure described by Brodinová et al. (2019). Each dataset has $n$ samples characterized by $p$ features, with $p=p_{d}+p_{n}$, where $p_{d}$ is the number of discriminant features and $p_{n}$ is the number of uninformative features. Each sample comes from one of $C$ clusters. Let $n_{t}$ be the size of the cluster $t$, and we have $n=\sum_{t=1}^{C} n_{t}$. In this study, we choose $p_{d}=50$, $p_{n}=950$, $C=3$, and $n_{t}=40$ for $t=1, 2, 3$.

Samples of each cluster are fully characterized by the discriminant features, which follow a multivariate normal distribution with a mean vector $\mu_{t}\in\mathbb{R}^{p_{d}}$ and a covariance matrix $\Sigma_{t}\in\mathbb{R}^{p_{d}\times p_{d}}$, for $t=1,\ldots,C$. The elements of the mean vector $\mu_{t}$ are constructed as

$$\mu_{tj}=\left\{ \begin{aligned} \mu, &j=mC+t \\ 0, &\mathrm{otherwise} \end{aligned} \right.$$

where $1\leq j\leq p_{d}$, $\mu$ is a constant, and $m$ is any nonnegative integer. The larger $\mu$, the farther away the clusters are located geometrically. The covariance matrix $\Sigma_{t}$ is constructed as

$$\Sigma_{t}=\mathbf{Q}\left( \begin{matrix} 1 & \rho_{t} & \ldots& \rho_{t} \\ \rho_{t} & \ddots& \ddots& \vdots\\ \vdots& \ddots& \ddots& \rho_{t} \\ \rho_{t} & \ldots& \rho_{t} & 1 \end{matrix} \right)\mathbf{Q}^{T},$$

where $\mathbf{Q}$ is a random orthogonal matrix and $\rho_{t}$ is a random number sampled from a uniform distribution $U\left( a_{\rho},b_{\rho} \right)$ with $0<a_{\rho},b_{\rho}<1$. Here, we choose $\mu=2$, $a_{\rho}=0.1$, and $b_{\rho}=0.9$ to generate clusters that are close to each other, rendering a nontrivial problem for sparse clustering algorithms. For the uninformative features of each sample, we generate $p_{n}$ random numbers that are independently and identically distributed from the standard normal distribution.

**Benchmarking of sparse hierarchical clustering algorithms using simulation data**

Using the above procedure, we randomly generated 100 simulation datasets for benchmarking performances of the presented algorithms. In all the analyses, the dissimilarity metric between features was the squared distance, and the hyperparameters were chosen based on gap statistic profiles. In the SHC-SSL analyses, we set $\lambda_{1}=0.001$ and chose the optimal $\lambda_{0}$ based on gap statistic profiles.

To evaluate the performances of the presented algorithms, we used the following performance metrics.

*Recovery of discriminant features.* To evaluate feature selection, given $\mathbf{w}$ from the result of the algorithms, we regard features $\left\{ j|w_{j}>0 \right\}$ as predicted discriminant features, and all the rest of features are predicted uninformative features. By comparing them with the true discriminant and uninformative features, we obtain precision, recall, and F1 score of the discriminant features.

*Silhouette.* To measure the clustering effect, we calculate the silhouette value using the dissimilarity matrix $\mathbf{U}$ and true cluster labels of samples. We use the silhouette value to measure the consistency of $\mathbf{U}$ from the true clusters rather than the consistency of a predicted partition from a fixed dissimilarity matrix.

*Concordance of clusters along dendrogram after hierarchical clustering.* The resulting dissimilarity matrix $\mathbf{U}$ is subjected to hierarchical clustering. To evaluate consistency of the resulting hierarchy of samples, represented as a dendrogram, with their true cluster labels, we calculate the concordance of cluster along the dendrogram as

$$\mathrm{Concordance}=\frac{\sum_{i=2}^{n} \mathbb{I}\left( L_{i}=L_{i-1} \right)}{n-C},$$

where $L_{i}$ is the label of the cluster to which the sample $i$ belongs. The denominator $n-C$ normalizes the range of the concordance to be within 0 and 1. The larger the concordance, the better the clustering. In the case of the worst clustering, when successive samples always belong to different clusters, the concordance becomes 0; in the case of an ideal clustering, when all within-cluster samples are tightly connected, the concordance is 1.

This metric measures a combined effect of hierarchical clustering and the chosen linkage criterion. Given $\mathbf{U}$, different linkage criteria generally produce different hierarchies. To compare different methods, we used the average linkage throughout this study.

*Adjusted Rand index and purity of partitioned clusters after Leiden clustering.* The resulting dissimilarity matrix $\mathbf{U}$ is used to find a shared $k$-nearest neighbor graph, where $k=20$. The neighbor graph is then subjected to Leiden clustering, in which the resolution parameter is set as 1, to partition samples into clusters. To evaluate consistency of the partitioned clusters with the true cluster labels, we calculate adjusted Rand index and purity. The larger these metrics, the better the clustering.

**Functional enrichment of discriminant markers and signature score calculation**

To relate RECOMBINE-selected discriminant markers with biological functions, we performed functional enrichment using Enrichr (Chen et al. 2013) with the MSigDB hallmark signatures (Liberzon et al. 2015) and WikiPathways (Kelder et al. 2012) provided by the Enrichr web server. Let’s denote $e_{ij}$ as the normalized and log-transformed expression of gene $j$ in cell $i$, and $G$ as the gene set of a signature. Following the signature score defined in Vision (DeTomaso et al. 2019), the signature score can be calculated as

$$s_{i}=\frac{\sum_{j\in G} e_{ij}}{\left| G \right|},$$

where $\left| G \right|$ denotes the size of the gene set $G$. Since discriminant markers can optimally distinguish cells, we calculate the signature score using only discriminant markers as

$$s_{i}=\frac{\sum_{j\in G} e_{ij}\mathbb{I}\left( w_{j}>0 \right)}{\sum_{j\in G} \mathbb{I}\left( w_{j}>0 \right)},$$

where $\mathbb{I}\left( w_{j}>0 \right)$ is an indicator function of gene $j$ selected in discriminant markers. As reported by DeTomaso et al. (2019), even if the expression values are normalized, the signature scores calculated as described above may still depend on technical variability across cells. To remove this variability, the signature score can be normalized using the expected mean and variance of the score of a random gene set having the same number of genes. For a random gene set, the expected mean of the signature score in cell $i$ is

$$E\left[ s_{i} \right]=\overline{e}_{i},$$

where $\overline{e}_{i}$ is the mean of expressions in cell $i$, and the expected variance of the signature score is

$$\mathrm{Var}\left[ s_{i} \right]=\frac{\sigma_{i}^{2}}{m},$$

where $\sigma_{i}^{2}$ is the variance of the expressions in cell $i$, and $m=\sum_{j\in G} \mathbb{I}\left( w_{j}>0 \right)$. Finally, the signature score is normalized as

$$s_{i}\leftarrow\frac{s_{i}-E\left[ s_{i} \right]}{\sqrt{\mathrm{Var}\left[ s_{i} \right]}}.$$

**References**

Brodinová Š, Filzmoser P, Ortner T, Breiteneder C, Rohm M. 2019. Robust and sparse k-means clustering for high-dimensional data. *Adv Data Anal Classif* **13**: 905–932. doi:10.1007/s11634-019-00356-9

Chen EY, Tan CM, Kou Y, Duan Q, Wang Z, Meirelles GV, Clark NR, Ma’ayan A. 2013. Enrichr: interactive and collaborative HTML5 gene list enrichment analysis tool. *BMC Bioinformatics* **14**: 128. doi:10.1186/1471-2105-14-128

DeTomaso D, Jones MG, Subramaniam M, Ashuach T, Ye CJ, Yosef N. 2019. Functional interpretation of single cell similarity maps. *Nat Commun* **10**: 4376. doi:10.1038/s41467-019-12235-0

Kelder T, van Iersel MP, Hanspers K, Kutmon M, Conklin BR, Evelo CT, Pico AR. 2012. WikiPathways: building research communities on biological pathways. *Nucleic Acids Res* **40**: D1301–D1307. doi:10.1093/nar/gkr1074

Liberzon A, Birger C, Thorvaldsdóttir H, Ghandi M, Mesirov JP, Tamayo P. 2015. The molecular signatures database hallmark gene set collection. *Cell Syst* **1**: 417–425. doi:10.1016/j.cels.2015.12.004

Liu J, Yuan L, Ye J. 2010. An efficient algorithm for a class of fused lasso problems. In *Proc 16th ACM SIGKDD Int Conf Knowl Discov Data Min*, pp. 323–332. doi:10.1145/1835804.1835847

Ročková V, George EI. 2018. The spike-and-slab lasso. *J Am Stat Assoc* **113**: 431–444. doi:10.1080/01621459.2016.1260469

Ročková V. 2018. Bayesian estimation of sparse signals with a continuous spike-and-slab prior. *Ann Stat* **46**: 401–437. doi:10.1214/17-AOS1554

Tibshirani R, Walther G, Hastie T. 2001. Estimating the number of clusters in a data set via the gap statistic. *J R Stat Soc B* **63**: 411–423. doi:10.1111/1467-9868.00293

Tibshirani R, Saunders M, Rosset S, Zhu J, Knight K. 2005. Sparsity and smoothness via the fused lasso. *J R Stat Soc B* **67**: 91–108. doi:10.1111/j.1467-9868.2005.00490.x

Witten DM, Tibshirani R. 2010. A framework for feature selection in clustering. *J Am Stat Assoc* **105**: 713–726. doi:10.1198/jasa.2010.tm09415

Xin B, Kawahara Y, Wang Y, Gao W. 2014. Efficient generalized fused lasso and its application to the diagnosis of Alzheimer’s disease. In *Proc AAAI Conf Artif Intell* **28**(1). doi:10.1609/aaai.v28i1.8977
