# Supplementary material for: RECOMBINE identifies recurrent composite markers of cell types and states
Source: Genome Res. 2026 Jun;36(6):1221–37. doi: 10.1101/gr.280817.125 (PMC13262949; doi:10.1101/gr.280817.125)
Supplement: Supplement 2 [file Supplemental_Figures.docx]

**Supplemental Figures**

Supplemental Fig. S1. UMAPs of datasets used for benchmarking.

Supplemental Fig. S2. Benchmarking RECOMBINE relative to other methods.

Supplemental Fig. S3. RECOMBINE applied to zebrafish scRNA-seq and mouse cerebellum Slide-seq datasets.

Supplemental Fig. S4. RECOMBINE applied to scRNA-seq and STARmap data of mouse visual cortex.

Supplemental Fig. S5. RECOMBINE applied to scRNA-seq data of pan-cancer CD8 T cells.

Supplemental Fig. S6. RECOMBINE identifies concise yet discriminative markers of a rare cell subpopulation of mouse intestine.

Supplemental Fig. S7. RECOMBINE reveals expression programs underlying inter-tumoral heterogeneity across melanoma patients.

Supplemental Fig. S8. RECOMBINE reveals expression programs underlying intra-tumoral heterogeneity within individual patients with triple-negative breast cancer.


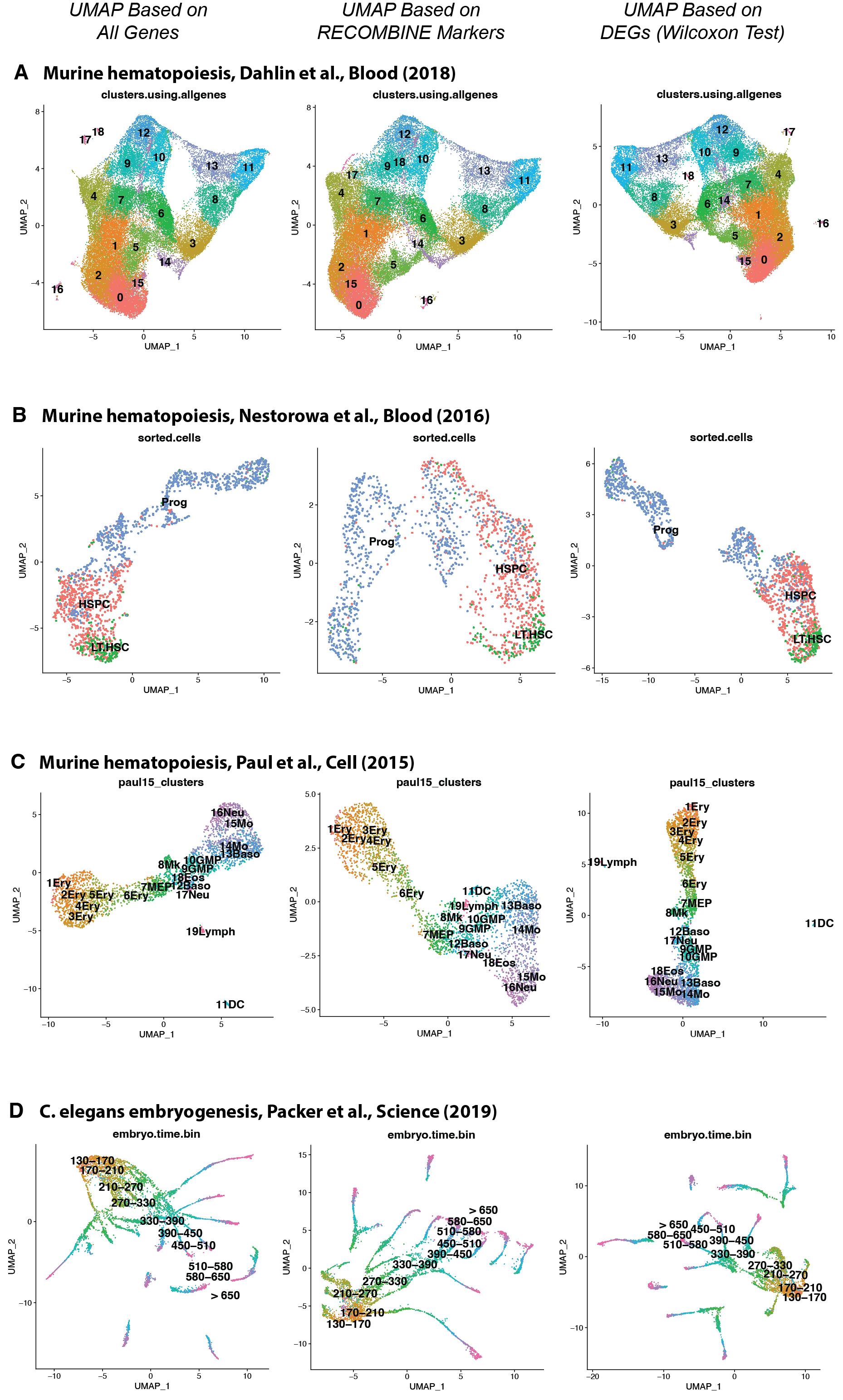


**Supplemental Fig. S1. UMAPs of datasets used for benchmarking. (A–D)** Four biological datasets. For each dataset (from left to right), UMAPs were generated using all genes, RECOMBINE-selected markers, and the top DEGs matched in number to the RECOMBINE markers.


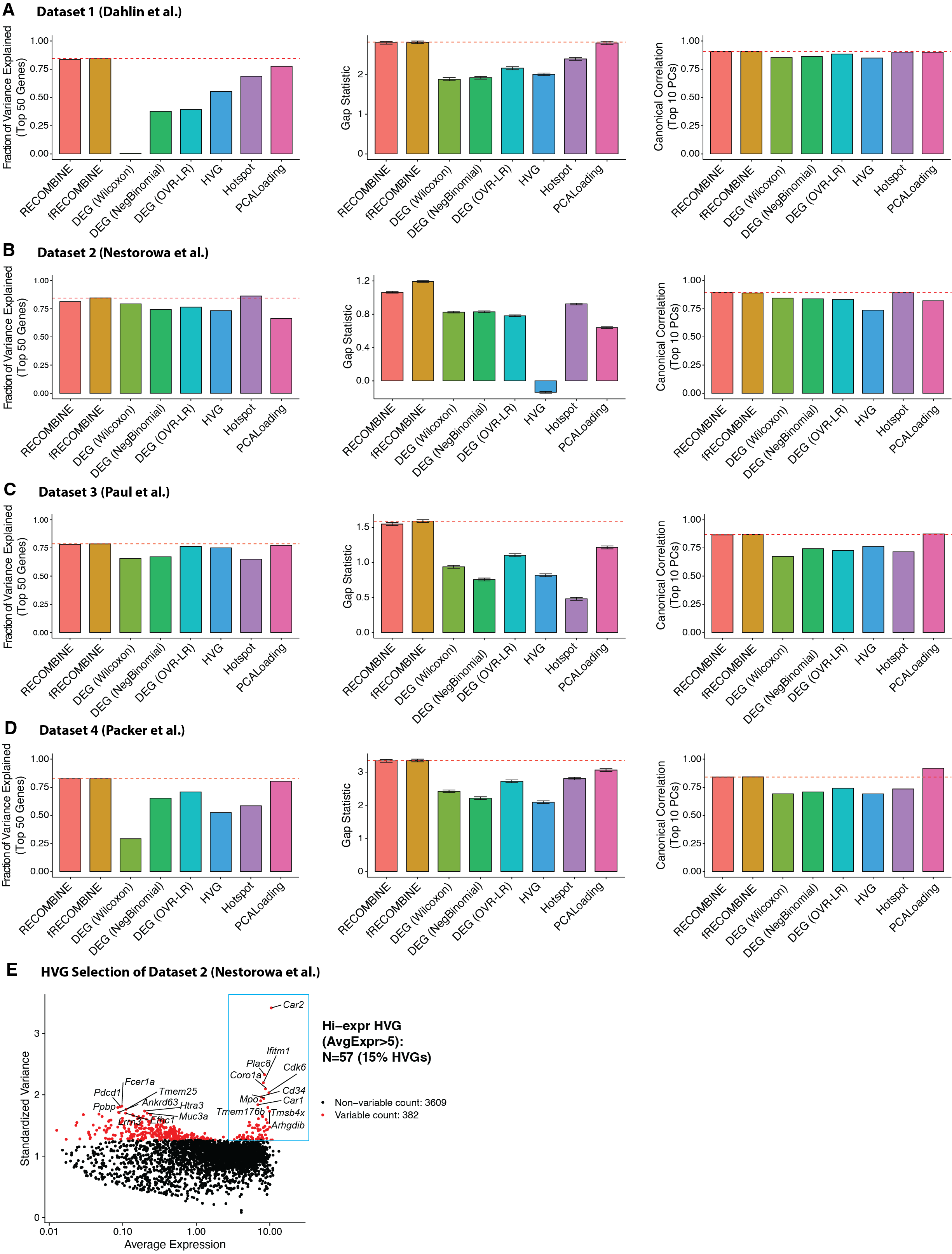


**Supplemental Fig. S2. Benchmarking RECOMBINE relative to other methods. (A–D)** Benchmarking results across four biological datasets. Left: Fraction of variance explained by the top 50 genes identified by each method, expressed as a proportion of the total variance captured by the top 10 principal components of the complete dataset (using all genes). Middle: Cell hierarchy discriminability (gap statistic), defined as the difference in hierarchical clustering strength between selected genes and randomly selected genes of equal number. For each method, the top features were selected to match the size of the RECOMBINE marker set (see Methods). Because fRECOMBINE is robust to feature ranking, it achieves gap statistics comparable to those of RECOMBINE. Notably, although fRECOMBINE shows improved gap statistics in Dataset 2, the evaluation was performed using the same marker set size (N = 382) identified by RECOMBINE, whereas fRECOMBINE yields a total of 3,981 markers with non-zero weights. These results suggest that fRECOMBINE is well suited for feature ranking, whereas RECOMBINE optimizes marker set size. Right: Canonical correlation between the top 10 principal components derived from selected genes and those from the complete dataset. **(E)** Mean–variance plot used in HVG selection for Dataset 2 (Nestorowa et al.) shows that only 57 genes (15% of HVGs) exhibit high mean expression. Because most HVGs have low expression levels and are susceptible to noise, the gap statistic of HVGs (B, middle) is lower than that of randomly selected genes.


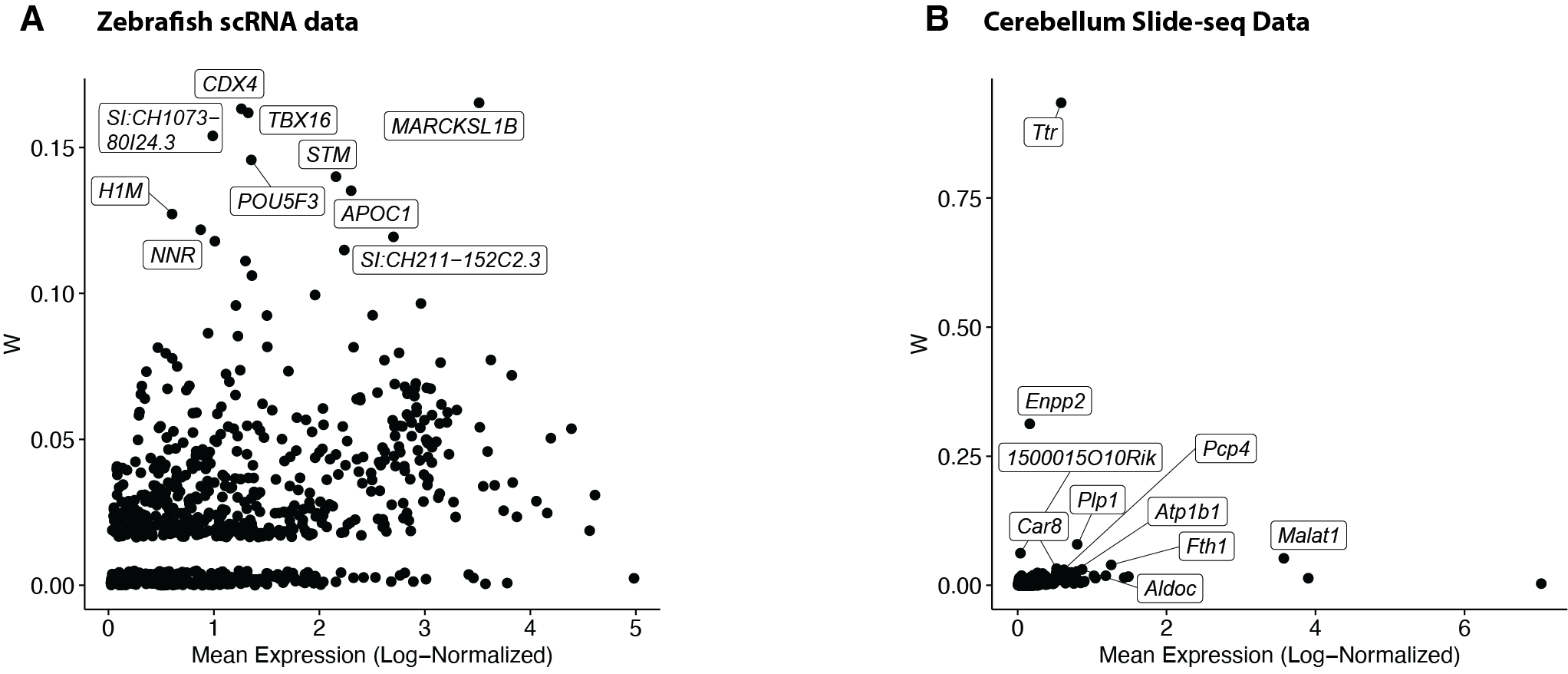


**Supplemental Fig. S3. RECOMBINE applied to zebrafish scRNA-seq and mouse cerebellum Slide-seq datasets.** Scatter plots showing RECOMBINE feature weights versus mean log-normalized expression of discriminative features in zebrafish scRNA-seq data **(A)** and mouse cerebellum Slide-seq data **(B)**.


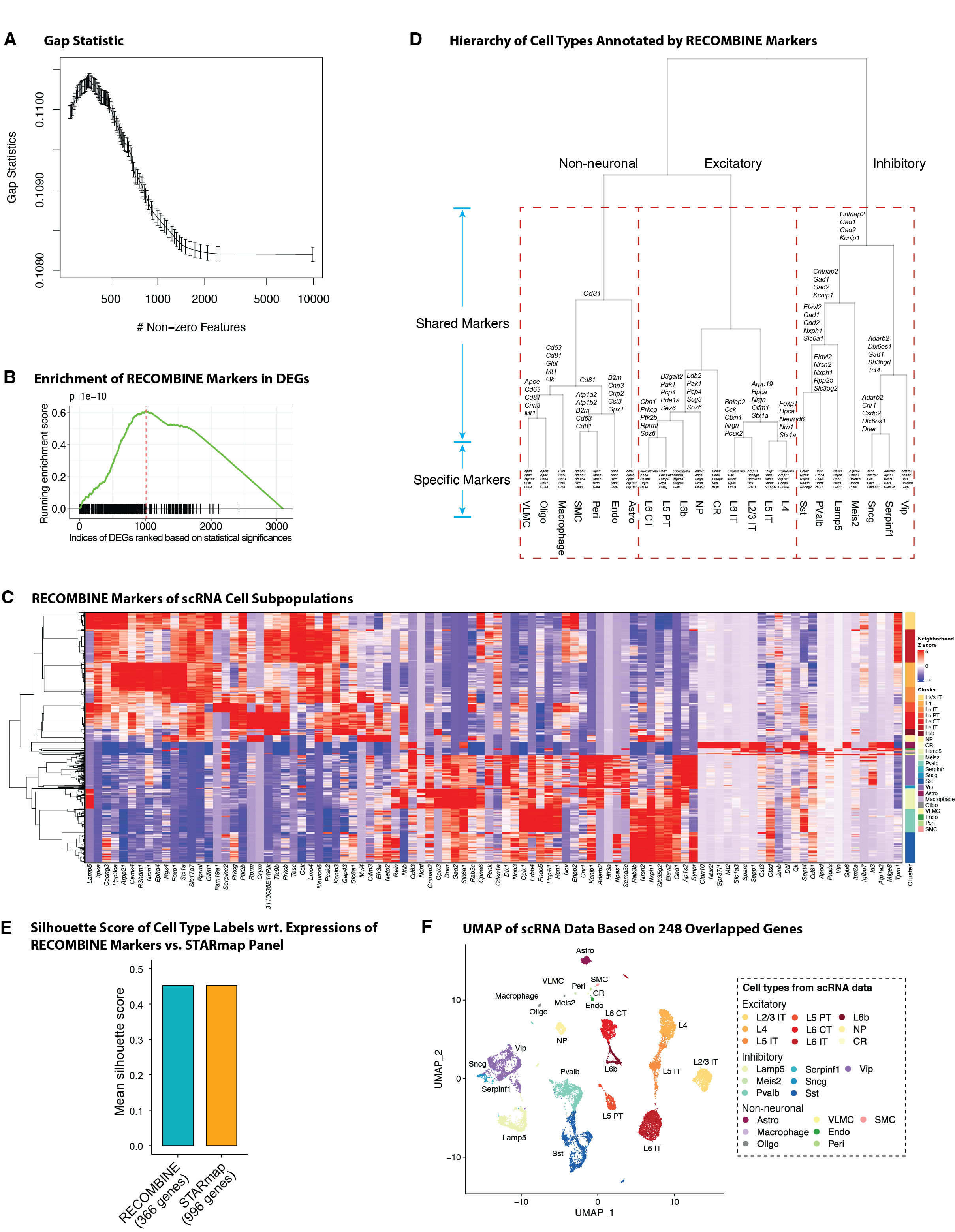


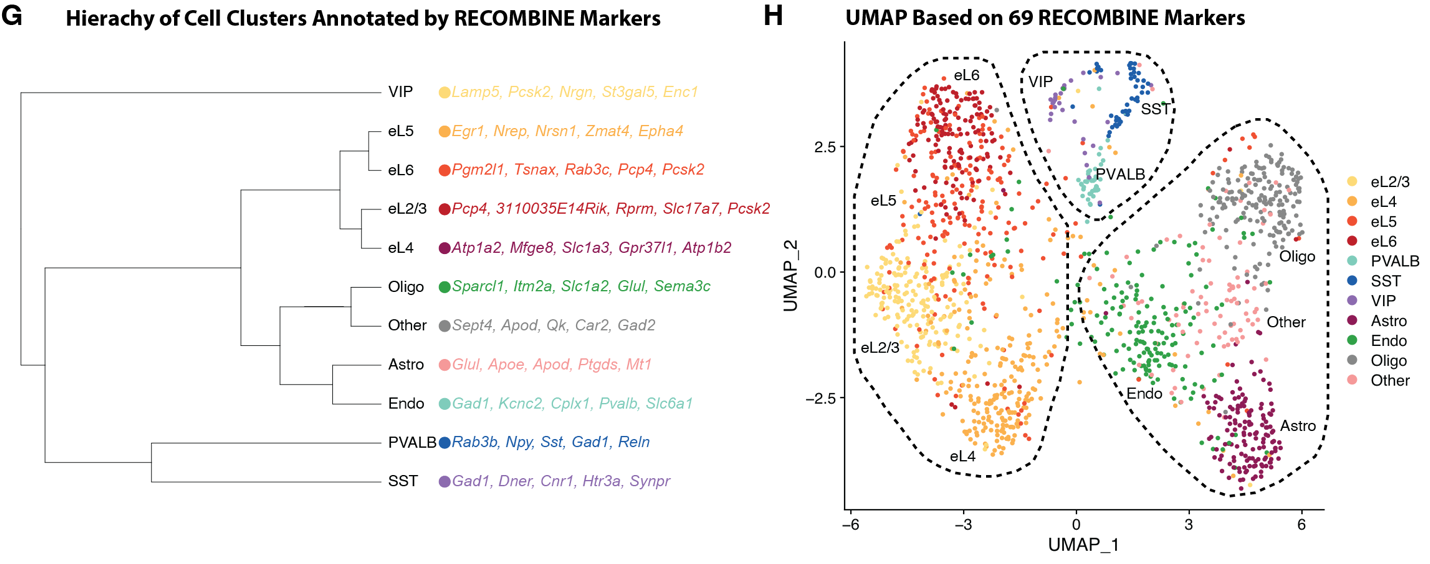


**Supplemental Fig. S4. RECOMBINE applied to scRNA-seq and STARmap data of mouse visual cortex. (A)** Gap statistic as a function of the number of selected genes. **(B)** Enrichment of RECOMBINE markers within the DEGs ranked by their significance levels (Wilcoxon test). **(C)** Heatmap of neighborhood *Z* scores showing gene modules across cell types of the scRNA data. **(D)** Hierarchical structure of cell types with each node annotated by RECOMBINE markers. **(E)** Silhouette scores of cell type labels based on the expression of RECOMBINE markers versus the STARmap gene panel using scRNA-seq data. With only 366 genes, RECOMBINE markers achieve comparable performance to the 996-gene STARmap panel. **(F)** UMAP of cells based on the 248 RECOMBINE markers overlapping with the STARmap panel. **(G)** Hierarchy of cell clusters annotated by top 5 RECOMBINE markers. **(H)** UMAP of spatially resolved cells based on a reduced-size panel of RECOMBINE markers (N=69), representing the top five genes per cluster with mean neighborhood *Z* score > 2, ranked by decreasing fraction of significant cells.


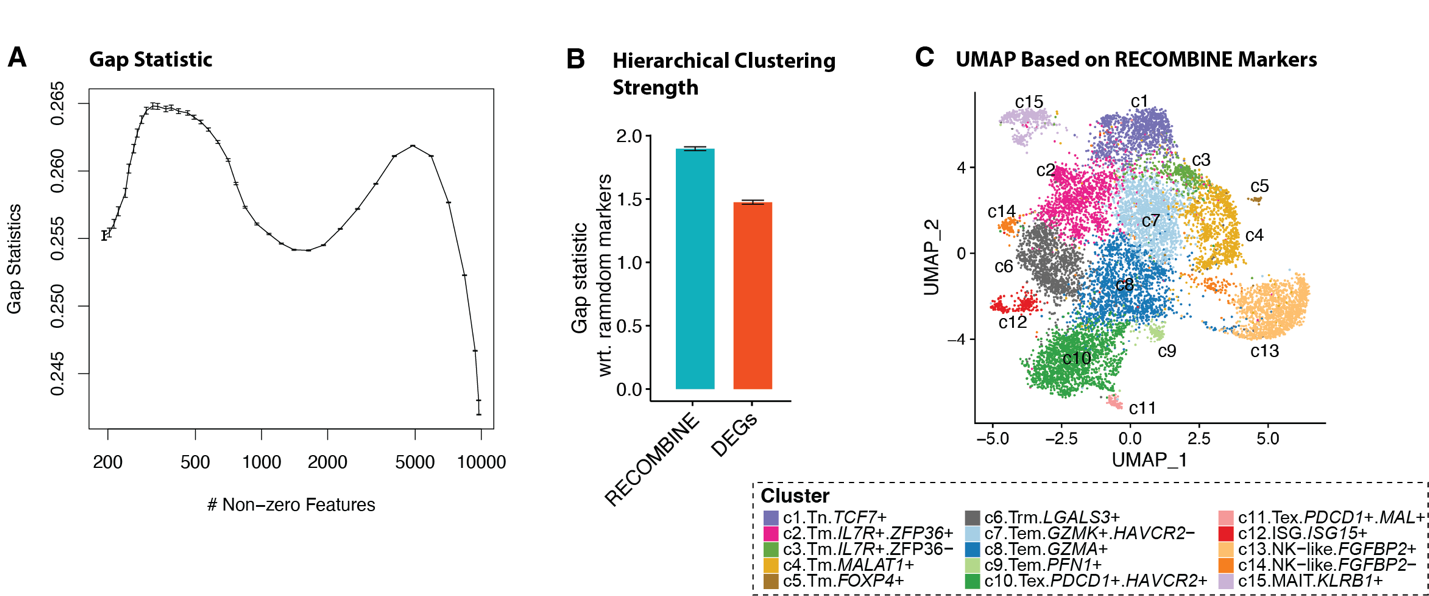


**Supplemental Fig. S5. RECOMBINE applied to scRNA-seq data of pan-cancer CD8 T cells. (A)** Gap statistic as a function of the number of selected genes. **(B)** Comparison of hierarchical clustering performance between RECOMBINE markers and top DEGs of the same size. **(C)** UMAP based on RECOMBINE markers and colored by clusters.


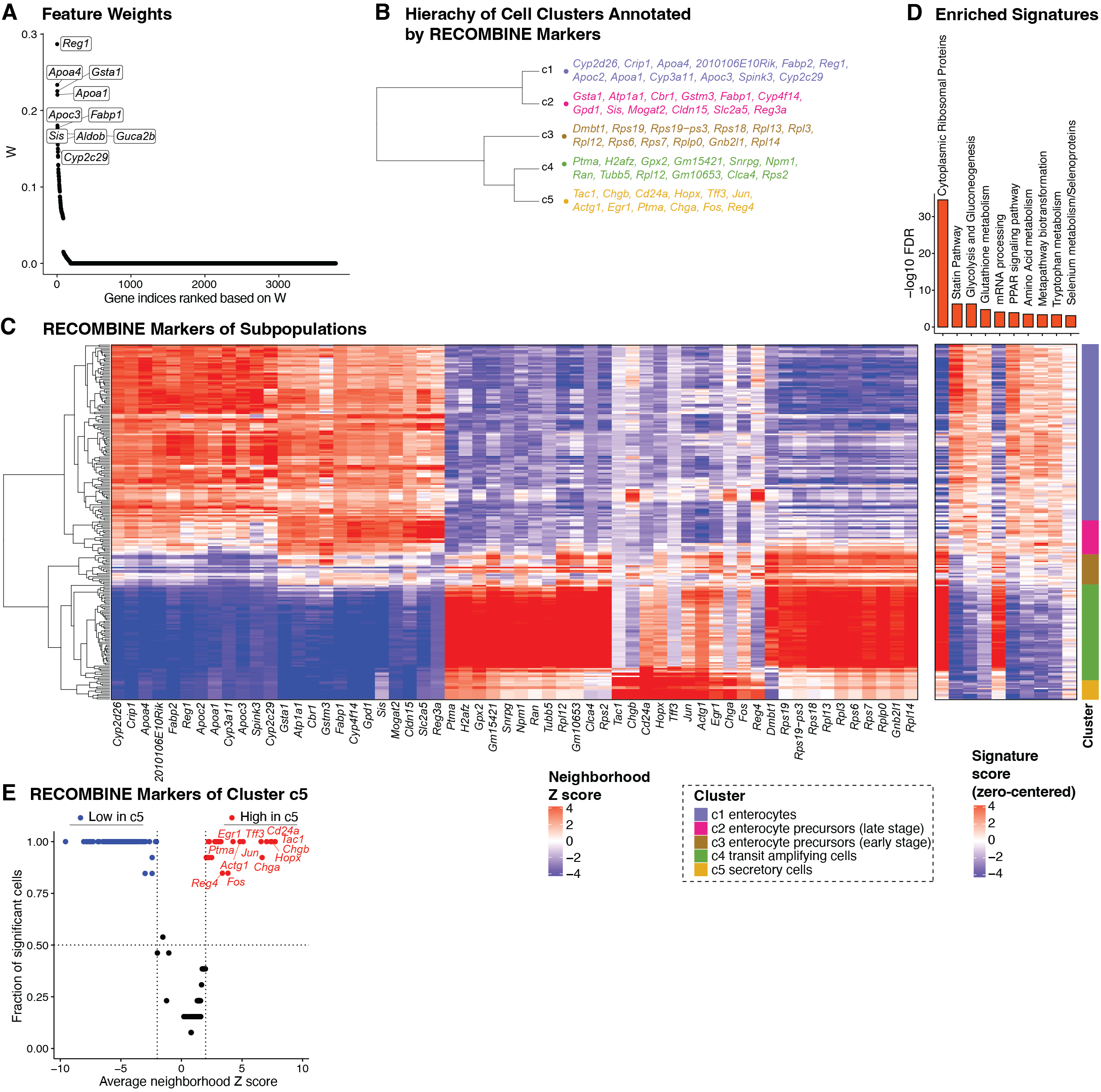


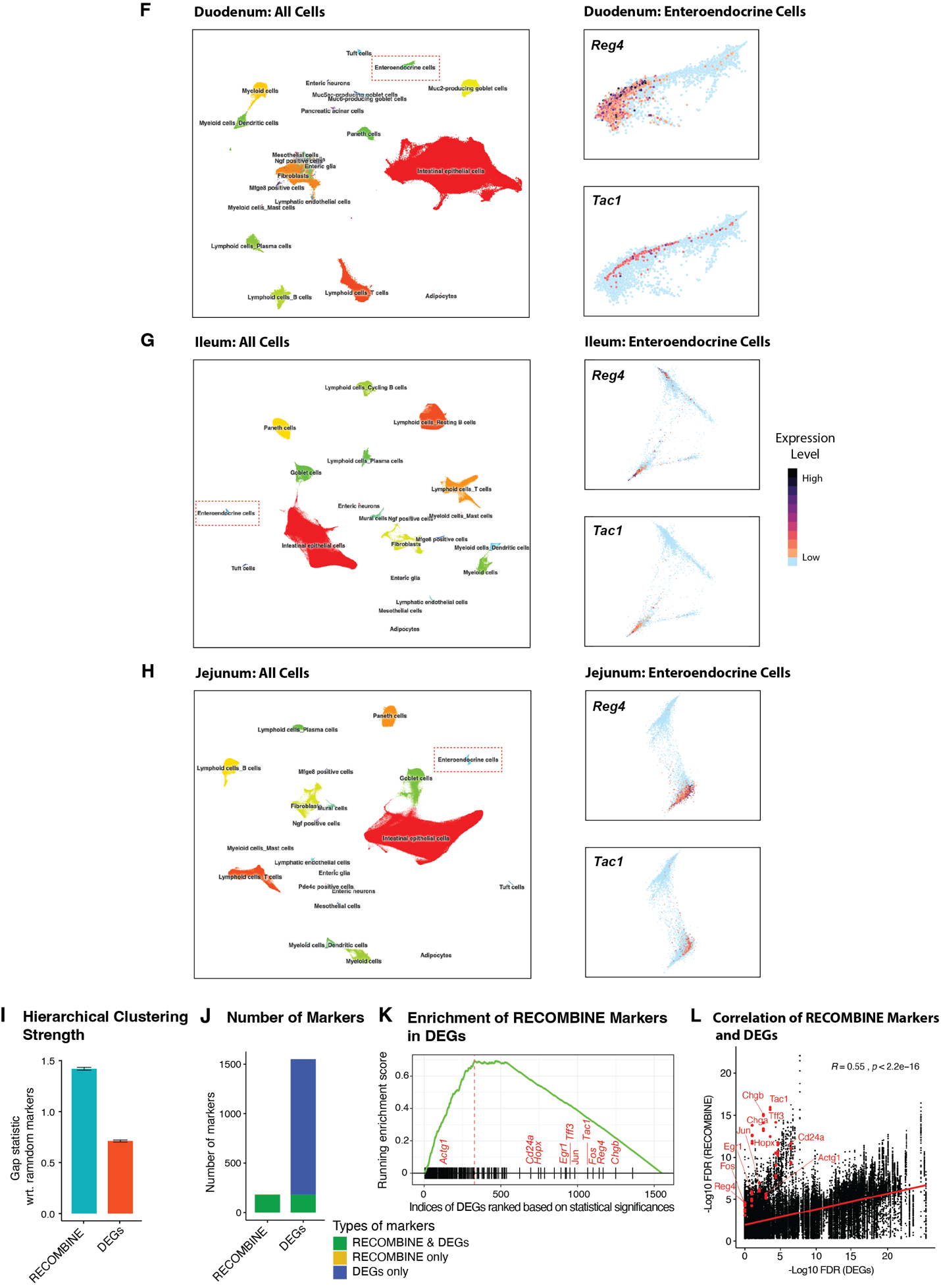


**Supplemental Fig. S6. RECOMBINE identifies concise yet discriminative markers of a rare cell subpopulation of mouse intestine. (A)** Feature weights of all genes where the top 10 discriminant markers are labeled. **(B)** Hierarchy of clusters annotated by top 10 RECOMBINE markers. **(C)** Heatmap of neighborhood Z scores showing gene modules across clusters. **(D)** Enrichment of mouse pathway signatures in RECOMBINE markers (top) and heatmap of signature scores across cells (bottom). Top 10 significantly enriched signatures are shown. **(E)** Volcano plot of cluster c5-specific markers, highlighting markers of a rare cell subpopulation in red. **(F-H)** Validation of the rare Reg4⁺/Tac1⁺ enteroendocrine cell population in the duodenum, ileum, and jejunum using data from PanSci, a mouse aging cell atlas (Zhang et al.). **(I)** Comparison of hierarchical clustering performance between RECOMBINE markers and top DEGs of the same size. **(J)** Comparison of RECOMBINE markers and DEGs. DEGs were obtained by Leiden clustering of all cells based on all genes followed by Wilcoxon test for each cluster with respect to the rest of the clusters. RECOMBINE markers were statistically significant markers with false discovery rate (FDR) < 0.05, and DEGs were filtered based on absolute log2 fold-change > 0.25 and FDR < 0.05. **(K)** Enrichment plot of RECOMBINE markers in the list of DEGs ranked by their significance from Wilcoxon tests. **(L)** Comparison of FDRs between RECOMBINE and DEGs for all cells. As FDRs of DEGs were obtained at the cluster level, we used the same FDRs for cells within the same cluster. The rare Reg4^+^ subpopulation’s markers of cluster c5 are highlighted in red.


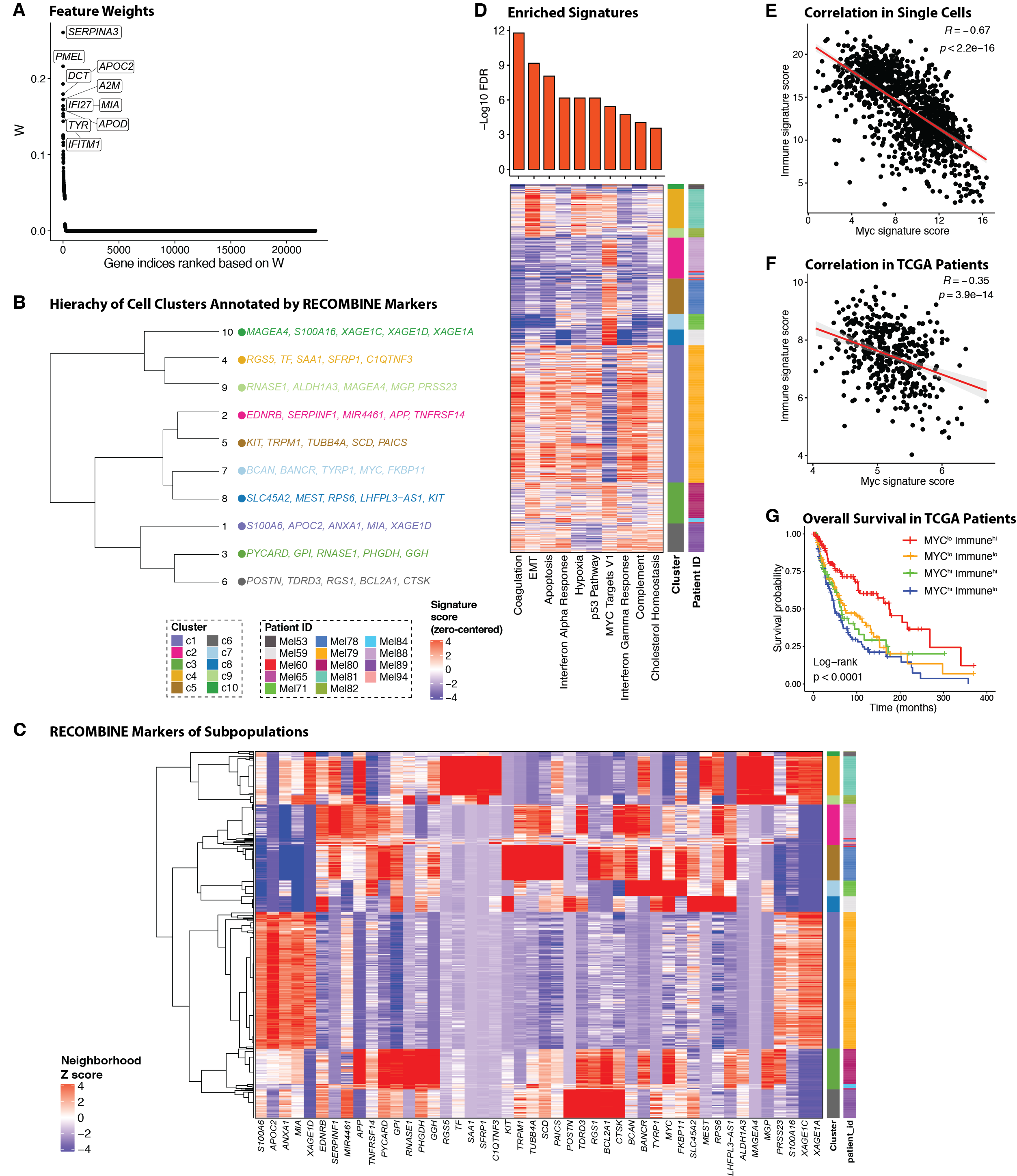


**Supplemental Fig. S7. RECOMBINE reveals expression programs underlying inter-tumoral heterogeneity across melanoma patients.** **(A)** Feature weights of all genes, with the top 10 discriminant markers labeled. **(B)** Hierarchical tree of clusters annotated by the top five RECOMBINE markers. **(C)** Heatmap of neighborhood *Z* scores showing RECOMBINE gene modules across clusters. **(D)** Enrichment of MSigDB hallmark signatures among RECOMBINE markers (top) and heatmap of signature scores across cells (bottom). The top 10 significantly enriched signatures are shown. **(E)** Correlation between MYC and immune signature scores across cells, with a red line indicating linear regression. **(F)** Correlation between MYC and immune signature scores computed from bulk mRNA expression profiles across 443 melanoma patients (TCGA). **(G)** Overall survival of TCGA melanoma patients stratified by MYC and immune signature scores.


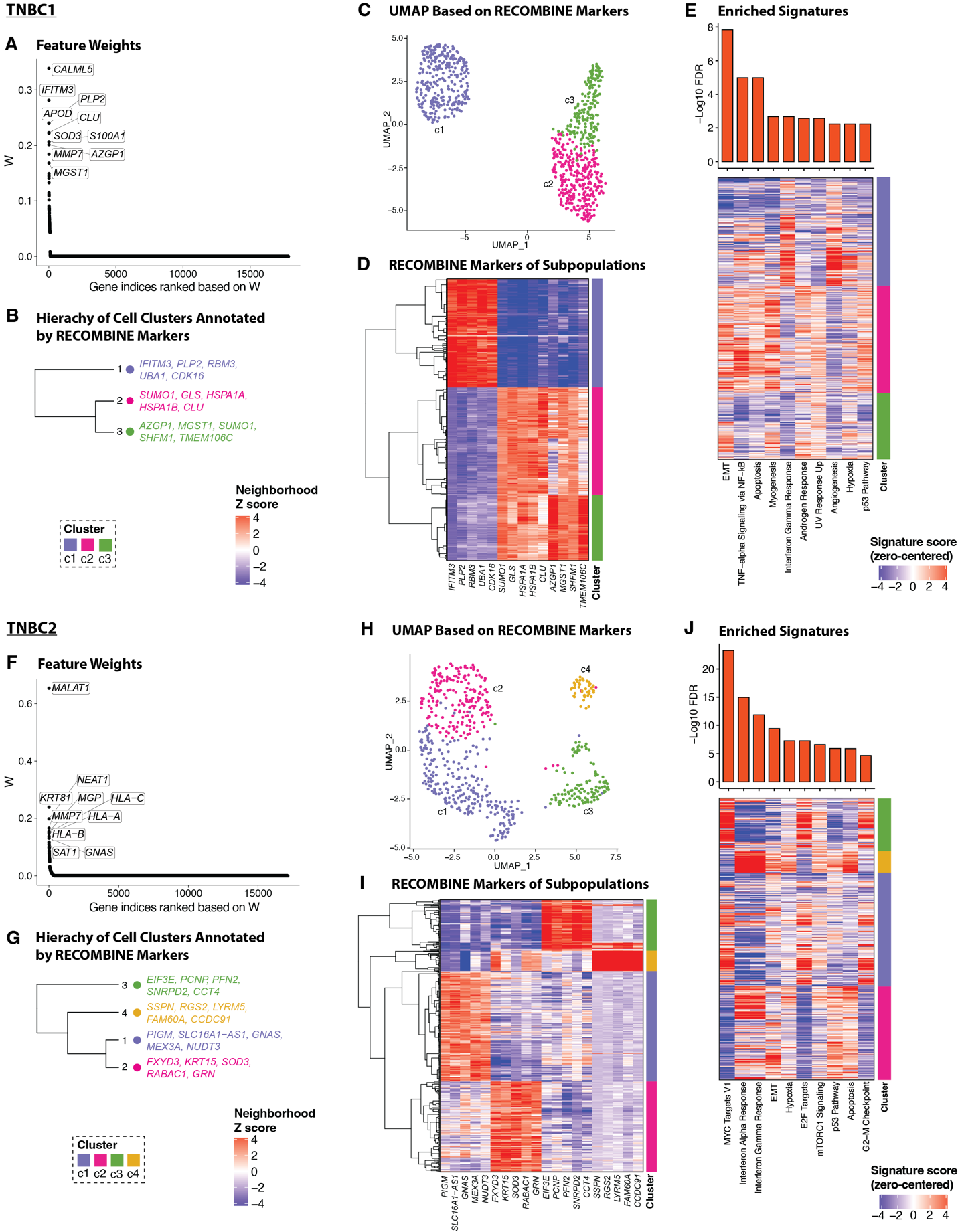


**Supplemental Fig. S8. RECOMBINE reveals expression programs underlying intra-tumoral heterogeneity within individual patients with triple-negative breast cancer (TNBC).** **(A–E)** Patient 1; **(F–J)** Patient 2. **(A, F)** Feature weights of all genes, with the top 10 discriminant markers labeled. **(B, G)** Hierarchical tree of clusters annotated by the top five RECOMBINE markers. **(C, H)** UMAP of cells based on RECOMBINE-selected markers, colored by cluster. **(D, I)** Heatmap of neighborhood *Z* scores showing RECOMBINE gene modules across clusters. **(E, J)** Enrichment of MSigDB hallmark signatures among RECOMBINE markers (top) and heatmap of signature scores across cells (bottom). The top 10 significantly enriched signatures are shown.
